# A new GTSeq resource to facilitate multijurisdictional research and management of walleye *Sander vitreus*

**DOI:** 10.1002/ece3.9591

**Published:** 2022-12-14

**Authors:** Peter T. Euclide, Wesley A. Larson, Matthew Bootsma, Loren M. Miller, Kim T. Scribner, Wendylee Stott, Chris C. Wilson, Emily K. Latch

**Affiliations:** ^1^ Department of Forestry and Natural Resources Purdue University West Lafayette Indiana USA; ^2^ College of Natural Resources University of Wisconsin‐Stevens Point Stevens Point Wisconsin USA; ^3^ National Marine Fisheries Service, Alaska Fisheries Science Center National Oceanographic and Atmospheric Administration Juneau Alaska USA; ^4^ Minnesota Department of Natural Resources St. Paul Minnesota USA; ^5^ Department of Fish and Wildlife Department of Integrative Biology Michigan State University East Lansing Michigan USA; ^6^ Department of Fisheries and Oceans, Artic and Aquatic Research Division Winnipeg Manitoba Canada; ^7^ Ontario Ministry of Natural Resources and Forestry Trent University Peterborough Ontario Canada; ^8^ Department of Biological Sciences University of Wisconsin‐Milwaukee Milwaukee Wisconsin USA

**Keywords:** amplicon sequencing, fisheries, Great Lakes, GTSeq, marker panel, mixed‐stock assignment, RAD capture

## Abstract

Conservation and management professionals often work across jurisdictional boundaries to identify broad ecological patterns. These collaborations help to protect populations whose distributions span political borders. One common limitation to multijurisdictional collaboration is consistency in data recording and reporting. This limitation can impact genetic research, which relies on data about specific markers in an organism's genome. Incomplete overlap of markers between separate studies can prevent direct comparisons of results. Standardized marker panels can reduce the impact of this issue and provide a common starting place for new research. Genotyping‐in‐thousands (GTSeq) is one approach used to create standardized marker panels for nonmodel organisms. Here, we describe the development, optimization, and early assessments of a new GTSeq panel for use with walleye (*Sander vitreus*) from the Great Lakes region of North America. High genome‐coverage sequencing conducted using RAD capture provided genotypes for thousands of single nucleotide polymorphisms (SNPs). From these markers, SNP and microhaplotype markers were chosen, which were informative for genetic stock identification (GSI) and kinship analysis. The final GTSeq panel contained 500 markers, including 197 microhaplotypes and 303 SNPs. Leave‐one‐out GSI simulations indicated that GSI accuracy should be greater than 80% in most jurisdictions. The false‐positive rates of parent‐offspring and full‐sibling kinship identification were found to be low. Finally, genotypes could be consistently scored among separate sequencing runs >94% of the time. Results indicate that the GTSeq panel that we developed should perform well for multijurisdictional walleye research throughout the Great Lakes region.

## Graphical abstract

New genetic marker panels are an important resource for wildlife management and conservation. Here, we develop and test a new GTSeq marker panel for walleye with Laurentian Great Lakes ancestry. The new panel provides accurate genetic stock and pedigree assignment for a large number of populations that will facilitate interjurisdictional research and collaboration.

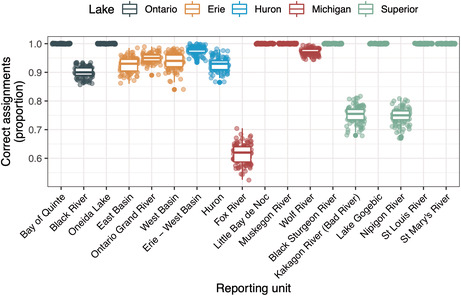

## 1 INTRODUCTION

Effective conservation of biological diversity requires collaborative research to inform conservation or natural resource planning. In many cases, this involves working across political boundaries and merging datasets generated in different laboratories to identify broad ecological patterns undetectable at a more regional scale (Jay et al., 2016; Margerum, 2008). Unfortunately, merging independent datasets is often impeded when studies do not share a common methodology (de Groot et al., 2015; Fairweather et al., 2018; Hunter et al., 2020). This can be an issue for genetic studies, which frequently generate marker sets de novo for each experiment (e.g., genotyping‐by‐sequencing; restriction site‐associated DNA sequencing [RAD‐seq]) or use laboratory‐specific protocols or marker panels (e.g., microsatellite genotyping) that result in genotype scoring discrepancies when datasets are merged (Goh et al., 2017; Pasqualotto et al., 2007). Without standardized methods and marker panels, genetic data generated from independent laboratories can be difficult or impossible to merge, limiting opportunities for collaboration and hampering the incorporation of molecular resources into natural resource planning.

Establishing standardized marker panels is important because genetic data provide insight into population biology and connectivity, recruitment dynamics, assessments of historical demography, and population‐specific mortality, which can take place across a large geographical area (Allendorf et al., 2010; Benestan et al., 2016). Therefore, collaboration among researchers is often necessary to extend population genetic research beyond a local scale (McKinney et al., 2020; Ruzzante et al., 1999). Historically, standardized marker panels for nonmodel species have mostly included microsatellite panels, or more recently, TaqMan assays, which require extensive laboratory validation to ensure genotype accuracy (Ellis et al., 2011; Hui et al., 2008; Seeb et al., 2007). Data collected using these types of resources have enabled managers to work collaboratively to inform policies structured around a species or population boundary, rather than a political or jurisdictional boundary (Homola et al., 2019; White et al., 2021). The development of new marker panels for common study organisms that are less reliant on intensive laboratory validation than microsatellite panels could benefit many species.

Standardized resources may particularly benefit the conservation of mobile species that frequently cross political boundaries (e.g., border waters of the Laurentian Great Lakes (Hildebrand et al., 2002) or transboundary conservation regions such as the Kavango–Zambezi Transfrontier Conservation Area (KAZA) in Africa or the Amazon River basin in South America; Mena et al., 2020; Stoldt et al., 2020). Species in these transboundary regions are often managed by multiple agencies that conduct research separately but must work collaboratively to protect the entire population. Sequencing‐based genotyping panels, such as genotyping‐in‐thousands (GTSeq), are becoming an increasingly accessible approach for nonmodel organisms (Campbell et al., 2015; Meek & Larson, 2019). Because this approach uses DNA sequencing, which provides exact nucleotide arrangements, the resulting genotypes can be more easily and consistently compared among studies than other PCR‐based assays. Other approaches such as microsatellite DNA markers, which require manual allele calling, are more vulnerable to human error and laboratory variability, making inter‐laboratory comparisons more difficult. The adoption of amplicon sequencing panels by laboratories with a purview of conducting research in major transboundary regions can help to facilitate collaboration and generate data that can be used for large‐scale meta‐analyses or long‐term monitoring of populations dynamics and genetic diversity (Hayward et al., 2022; McCane et al., 2018). However, published GTSeq panels are still unavailable for most species and can be time‐consuming to develop and implement.

Many of the developed GTSeq panels are for species of fisheries management interest, such as Pacific salmon (e.g., Chang et al., 2021; McKinney et al., 2020) and trout (Bohling et al., 2021). Another species with a recently developed GTSeq panel is walleye (*Sander vitreus*; Bootsma et al., 2020). Walleye is a highly mobile predatory species of fish native to North America, with an expansive endemic range spanning most of the United States and Canada (Figure S1–S8; Billington et al., 2011). There are many applications for a genetic panel for walleye, including tracking hatchery outplants, genetic‐informed domestication of aquaculture strains, population genetics, and genetic stock identification (GSI) of natural populations (Euclide, Robinson, et al., 2021). The GTSeq panel developed by Bootsma et al. (2020) was created specifically for walleye in inland lakes in the Mississippi River basin of Wisconsin and Minnesota (Bootsma et al., 2020, 2021). However, allele frequencies and genetic diversity differ between Mississippi River basin and Great Lakes walleye populations. Therefore, there has been some concern that an additional marker panel may be necessary to inform the conservation and management of walleye populations with broader Great Lakes ancestry.

Walleye stocks support extensive recreational and commercial harvest managed by numerous First Nation and tribal communities, Canadian provincial agencies, and eight American states surrounding the Great Lakes. Walleye can swim hundreds of kilometers per year, which means that walleye produced in one jurisdiction contributes to fishing opportunities in other jurisdictions (Brenden et al., 2015; Hayden et al., 2014; Matley et al., 2020). With so many sources of walleye recruitment and mortality, tracking walleye productivity in the Great Lakes has been a priority (Wills et al., 2020). Genetics is one effective method to track walleye productivity and stock connectivity; however, previous work has relied on microsatellite panels or large single‐use genotyping‐by‐sequencing studies (Chen, Euclide, et al., 2020; Garner et al., 2013), neither of which provide the compositional consistency necessary to merge datasets produced in different laboratories.

Here we describe the multi‐omic development and outline applications of a new GTSeq panel developed from 29 walleye spawning populations in the Great Lakes. The objectives of our study were to: (1) develop a general‐use GTSeq panel that includes genetic diversity from major walleye stocks in state, provincial, and tribal management jurisdictions in the Great Lakes, (2) evaluate the effectiveness of the panel to conduct mixed‐stock analysis and pedigree/kinship analysis throughout the Great Lakes and within each lake, and (3) quantify genotype call variation among laboratories.

## 2 METHODS

### 2.1 Study system and genetic diversity survey

The Laurentian Great Lakes is centrally located in the walleye species range and contain numerous and interconnected stocks of walleye that colonized the lakes following the last ice age from three different glacial refugia: the Mississippian, Atlantic, and Missourian (Stepien et al., 2009; Stepien & Faber, 1998). Walleye spawn on rocky reefs and in rivers throughout all five of the Great Lakes and are believed to exhibit moderate to strong natal spawning site fidelity (Chen, Ludsin, et al., 2020). Regionally, walleye spawning stocks range from highly productive naturally reproducing stocks, such as those in the West Basin of Lake Erie, to recovering or recovered stocks supported by fish hatcheries, such as those in northwestern Lake Superior (Vandergoot et al., 2010; Wilson et al., 2007). Sometimes both naturally reproducing and recovering stocks can be found within the same lake, such as the Ontario Grand River stock in Lake Erie (MacDougall et al., 2007). Therefore, to comprehensively survey walleye genetic diversity in the Great Lakes, it was important to include samples from as many known active walleye spawning stocks throughout the Great Lakes as possible. Samples of walleye fin clips and DNA were compiled from existing collections at collaborating institutions or collected for the purpose of this study during routine spawning stock assessments. All samples were collected between the years 2000 and 2019 from mature individuals during the spawning season at one of 29 known spawning sites (Table 1). Special attention was paid to sampling locations in Lake Erie where walleye abundance is high and genetic differences among spawning sites are low (Chen, Euclide, et al., 2020; Stepien et al., 2012) and to known stocking sources or receiving populations (i.e., Oneida Lake that is the stocking source for Lake Ontario and Lake Gogebic that was stocked with the ancestral Saginaw Bay walleye stock).

**TABLE 1:** Collection site number, name, and haplotype diversity estimates for the final GTSeq panel for walleye from the Great Lakes region of North America.

Site #	Population	*N*	Average number of alleles	Effective number of alleles	*H*_o_	*G*_IS_	Markers out of HWE
1	Erie‐Bournes Beach	14	2.27	1.75	0.47	−0.200	22
2	Erie‐Cattaragus Creek	18	2.34	1.75	0.38	0.021	21
3	Erie‐Chicken Island Reef	33	2.41	1.76	0.37	0.042	31
4	Erie‐Detroit River	61	2.50	1.79	0.40	−0.026	34
5	Erie‐Grand River, Ohio	13	2.27	1.76	0.40	−0.020	9
6	Erie‐Maumee River	73	2.54	1.79	0.42	−0.079	50
7	Erie‐Grand River, Ontario	60	2.44	1.73	0.36	0.017	37
8	Erie‐Sandusky River	71	2.52	1.80	0.41	−0.049	32
9	Erie‐Shorehaven	47	2.43	1.77	0.38	0.000	29
10	Erie‐Lackawanna Shoal	24	2.38	1.76	0.41	−0.064	30
11	Erie‐Tourssant Reef	36	2.46	1.79	0.44	−0.122	50
12	Erie‐Van Buren Bay	49	2.47	1.77	0.38	−0.002	24
13	Erie‐Zellerhouse Reef	47	2.45	1.77	0.38	0.016	31
14	Huron‐Moon River	14	2.19	1.67	0.37	−0.005	14
15	Huron‐Tittabawasee River	48	2.47	1.78	0.39	0.024	23
16	Michigan‐Fox River	44	2.42	1.74	0.38	0.014	24
17	Michigan‐Little Bay De Noc	41	2.33	1.67	0.35	0.018	20
18	Michigan‐Muskegon River	48	2.38	1.68	0.35	0.020	27
19	Michigan‐Wolf River	35	2.33	1.68	0.37	−0.017	20
20	Ontario‐Bay of Quinte	32	2.36	1.76	0.42	−0.075	31
21	Ontario‐Black River	21	2.27	1.69	0.35	0.039	20
22	Ontario‐Oneida Lake	13	2.10	1.60	0.32	0.028	8
23	St. Clair‐Clinton River	47	2.42	1.83	0.40	0.013	28
24	Superior‐Black Sturgeon River	37	2.35	1.66	0.36	−0.022	14
25	Superior‐Kakagon River (Bad River)	20	2.33	1.72	0.39	−0.012	18
26	Superior‐Lake Gogebic	16	2.21	1.63	0.33	0.055	15
27	Superior‐Nipigon Bay	31	2.32	1.68	0.33	0.097	38
28	Superior‐St. Louis River	30	2.31	1.71	0.36	0.043	29
29	Superior‐St. Marys River	46	2.44	1.74	0.37	0.027	32

*Note*: Sample size (*N*), average number of alleles, effective number of alleles, observed (*H*_O_), Nei's inbreeding coefficient (*G*_IS_), and the number of markers out of 500 significantly departed from HWE at an α of .05. Site numbers correspond to labels in Figure 1.

An initial genetic survey was conducted for walleye from the Great Lakes using a subset of 45 of the compiled samples (8–10 individuals from each Great Lake). Putative loci and genotypes were identified de novo using RAD‐sequencing. In brief, single nucleotide polymorphisms (SNPs) were identified by conducting *PstI* RAD‐sequencing (Ali et al., 2016). The program STACKS v2.3 (Rochette et al., 2019) was used to identify and genotype SNPs using the de novo pipeline, which was then filtered based on minor allele frequency (MAF). Following marker identification and de novo genotyping, an early draft of the walleye genome was obtained from the Great Lake Genomics Center at the University of Wisconsin‐Milwaukee. Therefore, the alignment position on the draft genome was identified using bowtie2 version 2.2.4 (Langmead & Salzberg, 2012) and used as a filter to limit the linkage disequilibrium among panel loci by removing loci in close proximity to one another (*personal communication* Aurash Mohaimani, Angela Schmoldt, and Rebecca Klaper, Great Lakes Genomics Center; Table S1–S8). A more detailed description of the RAD‐sequencing methods is outlined in Appendix S1.

**FIGURE 1:**
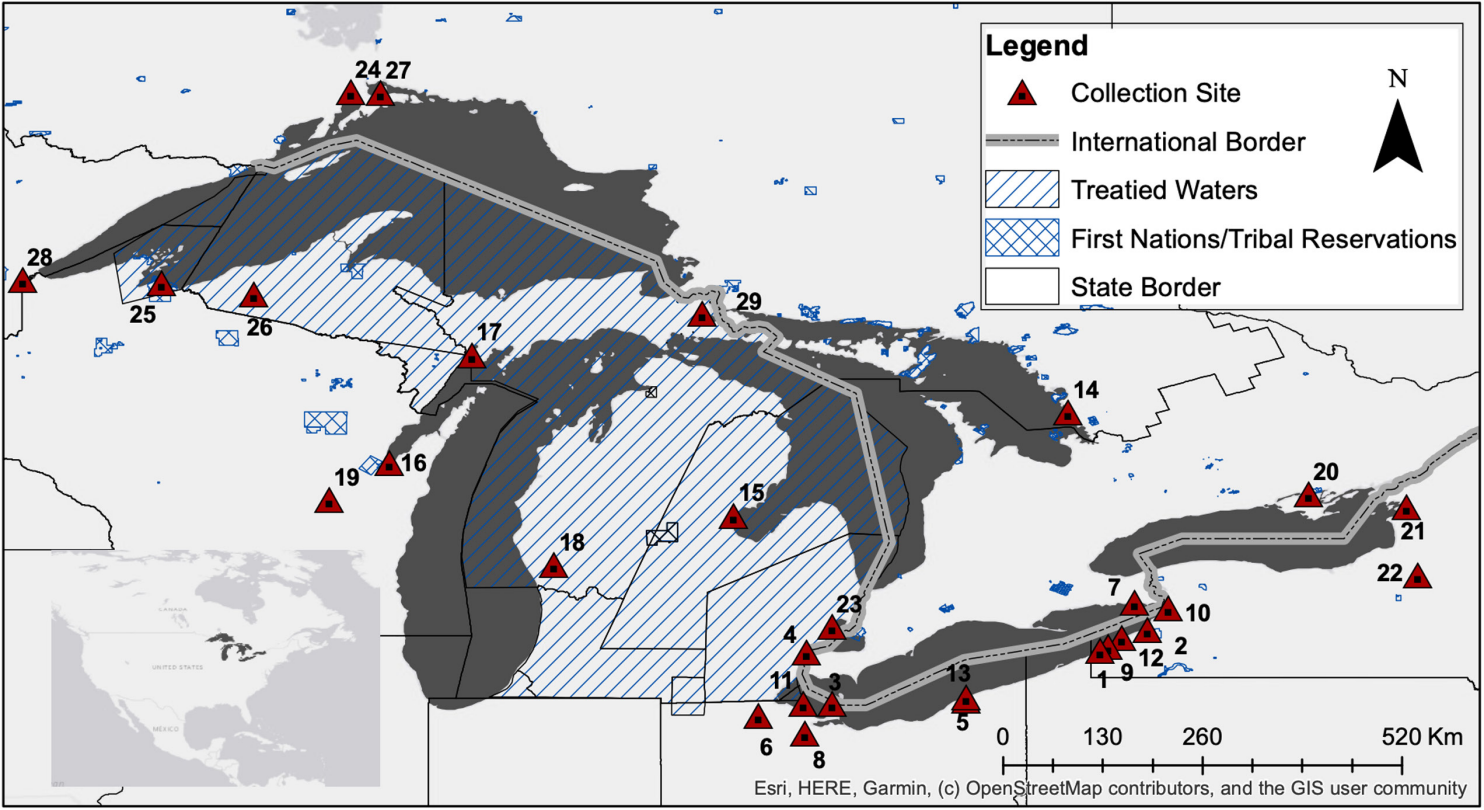
Geographical distribution of walleye spawning stock collection sites sampled for GTSeq panel design and major jurisdictional boundaries in the Great Lakes. Treated waters highlight regions with existing fishing access treaties between the United States and Great Lakes region tribal nations. Numbers correspond to site names listed in Table 1. Administrative boundaries were accessed from https://www.glahf.org/data/ December, 2021.

Following MAF and alignment position filters, 129,281 SNPs remained. Sequences for the 100,000 SNP loci, which contained the highest MAF and heterozygosity were submitted to ArborBioscience (Ann Arbor, MI) for capture bait development to create a Rapture panel. Capture baits were successfully designed for 99,636 loci (80 nucleotide baits with 2 × tiling). Sequencing libraries (maximum of 96 individuals per library) were then constructed for 1289 walleye spanning 29 walleye collection locations (Figure 1; Table 1) and bait captured following the approach outlined in Ali et al. (2016). These data were processed using STACKS v2.3 (Rochette et al., 2019) and quality filtered using the population step in STACKS v2.3 and VCFtools 2.3 (Danecek et al., 2011) to remove 220 individuals and 296,336 SNPs with poor genotyping rates (Table S2). Following filters, 44,261 of the baited loci with a genotype rate >70% across 1069 individuals were retained that were sequenced to an average depth of coverage of 19X.

### 2.2 Marker quality screening for GTSeq panel development

Microhaplotypes were identified and genotyped for all 44,261 SNP loci in the datafile using a whitelist containing marker IDs for each locus and the population module of STACKS v2.3. The resulting microhaplotype‐VCF file was then filtered using VCFtools to remove loci with >20% missing data (Danecek et al., 2011). Because the genotyping rate of microhaplotypes was lower than that of individual SNPs, to maintain a consistent set of loci in the final dataset, any microhaplotype removed due to missing data was replaced with the genotype of the individual SNP call with the highest minor allele frequency from the original 44,261 SNP datafile. Locus diversity was summarized with custom R scripts that used the DiveRsity and Adegenet R packages (Jombart, 2008; Keenan et al., 2013). Loci were sequentially removed as possible GTSeq panel candidates based on inbreeding coefficient (−0.2 < *F*_IS_ < 0.2), SNP position (17 < SNP position <140 on the forward read), and number of alleles per locus (<11). These filters removed 27,723 loci, leaving 16,538 as possible GTSeq panel candidates.

### 2.3 Marker selection scenarios

Our objective was to create a GTSeq panel containing 400 to 600 genetic markers. The number of potential markers was narrowed from 16,538 into five sets of 600 markers containing different numbers of markers that expressed high heterozygosity or allele frequency variance. High allele frequency differences (*F*_ST_; Weir & Cockerham, 1984) are important for applications such as GSI (e.g., Ozerov et al., 2013), while high heterozygosity can be important for kinship analysis (e.g., Baetscher et al., 2018; Blouin, 2003). The five scenarios included: (1) 600 loci with the highest *F*_ST_ and 0 markers chosen based on heterozygosity (FST600_mHE0); 450 loci chosen with the highest *F*_ST_ and 150 markers chosen based on heterozygosity (FST450_mHE150); 300 loci chosen with the highest *F*_ST_ and 300 markers chosen based on heterozygosity (FST300_mHE300); 150 loci chosen with the highest *F*_ST_ and 450 markers chosen based on heterozygosity (FST150_mHE450); 0 loci chosen with the highest *F*_ST_ and 600 markers chosen based on heterozygosity (FST0_mHE600). Marker sets were then subjected to GSI and kinship analysis simulations, and the panel mixture that performed well for both GSI and kinships was selected. GSI is frequently used for fisheries management to define management units and to track movement, and to assess contributions of different stocks to a mixed harvest while kinship analysis is the basis of many management‐focused activities, such as close‐kin mark‐recapture (CKMR) and parentage‐based tagging (Bravington et al., 2016; Schwartz et al., 2007). GSI simulations were conducted using Rubias (Moran & Anderson, 2019) and kinship simulations were conducted using CKMRsim following nearly identical protocols as outlined in Bootsma et al. (2020). The eight reporting units used for GSI simulations were defined based on prior knowledge of the system (i.e., existing jurisdictional and geographical breaks in the system) and included: Lake Ontario, the Ontario Grand River in Lake Erie, the East Basin of Lake Erie, the West Basin of Lake Erie, Lake Huron, Lake Michigan, the St. Mary's River, and Lake Superior (Figures S2 and S3). The FST450_mHE150 panel that showed intermediate performance was deemed the best general‐use selection scenario and used for subsequent panel design (see results; Figures S4 and S5).

### 2.4 Panel primer design

We selected 3X the number of loci in the 450:150 ratio of high *F*_ST_ to high microhaplotype heterozygosity for primer design to account for the loss of markers due to poor primer design. Primers were then designed for each marker using Primer3 v. 2.3 (Untergasser et al., 2012; Table S3). When more than one SNP was present at a locus, primers were designed to target as many SNPs as possible, but preference was given to the SNP with the highest minor allele frequency. However, if targeting the SNP with the highest minor allele frequency excluded three or more SNPs, primers were redesigned to exclude the highest minor allele frequency SNP and instead target the group of 3+ SNPs, thereby retaining the microhaplotype. Of the markers investigated, quality primer pairs were designed for 793 markers. Nine markers were removed due to potential off‐target amplification or identical forward and reverse primers. Diversity statistics were then used to select 600 markers from the remaining 784 markers to retain 450 markers originally selected based on SNP *F*_ST_ and 150 markers originally selected based on microhaplotype *H*_E_. The panel of 600 markers was then re‐assessed for GSI and parentage using identical protocols as preliminary screening to ensure that it performed similarly as the original FST450_mHE150 panel (Figures S4 and S5). Once satisfied, 6‐bp plate and sample adapters were added to forward and reverse primer sequences, and oligonucleotides for all 1200 primers were ordered from Integrated DNA Technologies (IDT, Coralville, Iowa).

### 2.5 Panel PCR optimization

The optimal multiplex combination of primer pairs was determined by conducting four sequential library preparation and sequencing runs on MiSeq Micro flow cells (paired‐end 150 bp; 300 cycles). A single library was run for each sequencing run. GTSeq libraries were prepared using the standard GTSeq library preparation protocols (Bootsma et al., 2020; Campbell et al., 2015). First, individuals were amplified in individual 7‐μl PCRs containing 1.5 μl multiplex primer mixture (final working concentration of 0.25 μM/primer), 3.5 μl Qiagen HotStar *Taq* Multiplex *Plus* DNA polymerase, primer mixture, and 2 μl DNA template. Next, plate (i7) and individual (i5) barcode adapters were ligated in a 10‐μL PCR‐containing 5 μl Qiagen HotStar *Taq* Multiplex *Plus* DNA polymerase, 1 μl of each i7 (10 μM) and 2 μl i5 barcodes (5 μM), and 2 μl of 3:17 diluted PCR product. The concentration of adapter‐ligated PCR product was normalized using SequalPrep Normalization plates (Applied Biosystems™) according to the manufacturer's protocol, pooled, and purified using a 0.65X followed by a 1.0X Beckman–Coulter Ampure bead cleanup and standard protocols outlined by Beckman‐Coulter. The amount of DNA in purified libraries was quantified using fluorometry on a Qubit (Life Technologies), and product size was assessed using an Agilent Bioanalyzer. Libraries that met quality checks (>0.1 ng/μl and correct product size) were loaded onto a MiSeq Micro flow cell (300 cycles) at 7 pM concentrations along with 10% PhiX spike (Illumina, Inc) and sequenced at either the University of Wisconsin Biotechnology Center (Optimization run 1) or the University of Wisconsin‐Milwaukee Great Lakes Genomics Center (Optimization runs 2–4). The data were demultiplexed by the sequencing core and sequencing reads associated with target SNPs were identified using the GTScore pipeline and the associated *AmpliconReadCounter* perl script and custom primer‐probe file (v.1.3; github.com/gjmckinney/GTscore). The results of each sequencing run were summarized in MS Excel and analyzed using custom R Scripts using (R v.4.1; R Core Team, 2021; Wickham, 2009; Xiao, 2018). Primers associated with overamplified sequences and primers producing a high number of primer dimers or off‐target reads (i.e., reads containing the primer sequence but not the target region) were removed iteratively in consecutive sequencing runs. Our target was to develop a mixture of primers that amplified a large number of markers evenly (i.e., similar depth of coverage across all markers). Therefore, we used the Shannon equitability index (*H*), which incorporates both richness (number of unique primer pairs remaining) and evenness of abundance (number of primer reads), as a measure of panel performance at each round of optimization. Values of *H* closer to 1 indicate a community with high evenness, therefore increases in *H* were defined as an increase in performance. The panel was deemed optimized once *H* was >0.8 (Table S4).

### 2.6 Final panel performance

Observed heterozygosity (*H*_O_), Nei's inbreeding coefficient (*G*_IS_; Nei, 1987), and Hardy–Weinberg equilibrium (HWE) was calculated for all markers in the final panel using Genodive v. 3 (Meirmans, 2020). Linkage disequilibrium among loci was measured by calculating the pairwise correlation coefficient (*r* ^2^) among all SNPs in the final panel using the SNPrelate R package (Zheng et al., 2012). Deviation from HWE was estimated using a one‐sided *t*‐test at an uncorrected alpha (α) of .05 and Bonferroni corrected α of .0001. We observed a high degree of spatial genetic structure among the collections used to develop the panel. Therefore, we expected the number of loci out of HWE and in linkage disequilibrium to be moderately high in the final panel.

The panel's utility for GSI and kinship was evaluated using data from the initial Rapture baseline filtered to retain only microhaplotype loci included in the final GTSeq panel. GSI performance was evaluated for two different sets of reporting units. First, sites were grouped by the lake to evaluate GSI among lakes, and then, sites were grouped within each lake to determine GSI within each lake. The sample size for Moon River in Lake Huron was small (*N* = 14) after removing individuals with a low genotyping rate; therefore, samples were combined with Tittabawassee samples to create a single Lake Huron reporting group. Additionally, because walleye from Lake St. Clair and western Lake Erie are known to mix with Lake Huron walleye (Brenden et al., 2015), samples from Lake St. Clair and the West Basin of Lake Erie were also included in the Lake Huron reporting group. Collections within all other lakes were analyzed separately. Pairwise Weir and Cockerham's *F*_ST_ was calculated among among‐lake and within‐lake reporting units as a metric of population structure (Meirmans, 2020; Weir & Cockerham, 1984).

Genetic stock identification simulations were conducted in Rubias using 99 replicate 100% leave‐one‐out simulations run using a mixture size of 200 individuals (Moran & Anderson, 2019). Expected assignment accuracy to collections within each lake was estimated using simulated sample mixtures in which 100% of the individuals are from one collection or reporting unit. Expected assignment accuracy was determined based on the number of individuals correctly assigned to their true collection location. A low group membership (*pofZ*) score of >0.7 was used to assign individuals to collections. The influence of *pofZ* thresholds of 0.7 to 0.95 on assignment results was evaluated by estimating the average assignment accuracy and number of unassigned individuals of each reporting unit at 0.05 *pofZ* intervals (Figure S6). The difference in average assignment accuracy of reporting units (*N* = 24) between a *pofZ* threshold of 0.7 and 0.95 was 1%. However, increasing *pofZ* to 0.95 led to a 1.8% increase in the number of unassigned individuals. Given the limited influence of *pofZ* threshold on the observed results, we chose to use a low *pofZ* threshold to retain as many assignment observations as possible. The proportion of correctly assigned individuals was calculated for each replicate and summarized to evaluate variance in assignment accuracy among all five lakes and among sampled spawning stocks within each lake. Stocks with high rates of misassignments (>10% on average) were investigated to determine where individuals were being misassigned.

Kinship simulations were conducted in CKMRSim independently within each lake using whole‐lake compositions of allele frequency to assess the power for pairwise kinship inference. The R package CKMRsim (https://github.com/eriqande/CKMRsim) uses a Monte Carlo sampling approach and importance‐sampling to make pairwise relationship inferences based on multiallelic data (see Baetscher et al., 2019 for additional descriptions). By simulating sets of related and unrelated pairs of individuals based on provided allele frequency data, an expected log‐likelihood ratio distribution of pairwise comparisons of individual kinship for different familial relationships (i.e., full‐sibling, half‐sibling, parent‐offspring, or unrelated) is created. Overlap in log‐likelihood ratio distributions can then be used to estimate false‐positive rates (FPR) and false‐negative rates (FNR) of relationship assignments based on a given set of markers and allele frequencies. Because false‐positive rates can greatly influence CKMR analyses (Bravington et al., 2016; Waples & Feutry, 2022), estimates of error rates help to determine whether a set of markers has sufficient power to assign pairwise relationship status between two individuals while minimizing false‐positive relationship assignment (i.e., assigning two individuals as related when they are in reality unrelated). False‐positive detection rates were calculated by using allele frequency data from each lake to simulate 1000 full‐sibling (FS), half‐sibling (HS), parent‐offspring (PO), and unrelated (U) pairs (4000 total). Next, the log‐likelihood of relatedness for a given pair of individuals was calculated for simulated related individuals. The relationship between observed genotype pair probabilities calculated for true related individuals (FS, HS, PO) was then compared with the hypothesis of no relationship (U). These values then were used to calculate distributions of log‐likelihoods of relatedness and to compute false‐positive rates. False‐positive rates for parent‐offspring, full‐sibling, and half‐sibling relationships were estimated at false‐negative rates (per‐pair rate of truly related individuals being inferred to be unrelated) ranging from 0.01 to 0.1. A range of false‐negative rates is used as the acceptable ratio of false‐positive to false‐negative rates varies based on the research question. The results of this analysis were used as our measure of the GTSeq panel's ability to be used for kinship analysis, with lower probabilities of false‐positive errors indicating higher panel performance.

### 2.7 GTSeq genotyping performance

The sequencing consistency of the GTSeq panel genotyping was assessed by comparing genotypes from the same 95 individuals sequenced independently in three different laboratories: University Wisconsin—Milwaukee (Milwaukee, WI, USA), USGS Great Lakes Science Center (GLSC; Ann Arbor, MI, USA), and the Ontario Ministry of Natural Resources and Forestry (OMNRF) aquatic genetics lab at Trent University (Peterborough, ON, CA).

Laboratories each prepared a GTSeq library using in‐house protocols and reagents and sequenced the library on a single MiSeq Micro flow cell (paired‐end 150 reads; 300 cycles). Libraries were prepared at UWM and the GLSC using Small RNA Sequencing Primer adapters while sequencing at Trent was conducted using primers modified to include Nextera XT adapters. Sequencing data from all laboratories were processed identically using the GTScore pipeline to first produce summaries of the number of amplicon reads containing target amplicons (primers) and markers (probes) by individual and locus. Amplicon reads for each sample then were used to score genotypes for each locus based on the number of probe reads for each SNP and a maximum likelihood algorithm described in McKinney et al. (2018) that accounts for variance in allele dosage.

The consistency in sequencing output (i.e., amplification and subsequent sequencing of targeted genetic markers) among libraries prepared at the UWM, GLSC, and OMNRF laboratories separately was evaluated using the number of reads containing a primer sequence to the number of reads containing a probe sequence for a given marker (i.e., the exact 30‐bp sequence flanking a known SNP or microhaplotype). Individual coverage was calculated as the total number of reads containing sequence data for both the primer and probe for a given marker divided by 500 (the total number of markers included). For each marker and individual, the data were analyzed as a proportion of primer reads to probe reads (here forward referred to as *primer: probe* proportion). The consistency in this proportion among datasets was evaluated using pairwise Pearson's correlations of marker‐specific *primer: probe* proportion. Consistent amplification of the GTSeq panel was expected to result in a strong positive result and high correlation coefficient (*r* ^2^). The relative differences in the individual or marker sequencing variance among preparations are described using the standard deviation in *primer: probe* proportion.

Genotypes were defined as “congruent” between two datasets if the same alleles were scored in both cases of a pairwise assessment between laboratories for a given individual and locus. In other words, if individual‐X contained an AG heterozygote score in both the UWM and GLSC datasets, the genotype was considered “congruent” between these datasets. Congruency in scored genotypes among separate sequencing runs was evaluated in a pairwise fashion. First, individuals that lacked genotype calls at 50% or more of the GTSeq markers were removed from the analysis. Then, the percent of identical genotype calls (e.g., a call that is scored as a heterozygote in both datasets being compared) was calculated for individuals. One‐way Analysis of Variance (ANOVA) was used to test whether the average percent of congruent genotypes differed between laboratory pairs. We hypothesized that depth of coverage may influence genotype call accuracy, and therefore also tested whether average individual total read count across all three sequencing runs influence percent congruency using an ANOVA.

## 3 RESULTS

### 3.1 Panel selection

All five tested panel‐marker combinations performed similarly for GSI to eight putative reporting units and kinship assignment of parent‐offspring and full‐sibling pairs (Table 2). The FST_600_mHE0 panel performed the best for GSI (mean assignment accuracy = 92.7%) but worst for kinship analysis (full‐sibling FPR_(FNR=0.01)_ = 3.8 × 10^−15^). By contrast, the FST_0_mHE600 panel performed more poorly for assignment accuracy (mean assignment accuracy = 89.2%) and was the best for kinship analysis (full‐sibling FPR_(FNR=0.01)_ = 5.8 × 10^−24^). Based on these results, we chose one of the intermediate panels (FST_450_mHE150), which appeared to perform moderately well for both GSI (mean assignment accuracy = 91.1%) and kinship (full‐sibling FPR_(FNR=0.01)_ = 2.3 × 10^−18^).

**TABLE 2:** Mean estimated assignment accuracy across eight reporting units (Lake Ontario, the Ontario Grand River in Lake Erie, the East Basin of Lake Erie, the West Basin of Lake Erie, Lake Huron, Lake Michigan, the St. Mary's River, and Lake Superior) and the estimated false‐positive rate (FPR) of full‐sibling assignment used to compare between five potential panels at an accepted false‐negative rate (FNR) of 0.01.

Panel	High *F*_ST_ SNPs	High *H*_O_ microhaplotypes	Mean assignment accuracy	FPR_FNR=0.01_
FST_600_mHE0	600	0	92.7%	3.9 × 10^−15^
FST_450_mHE150	**450**	**150**	**91.1%**	**1.9 × 10** ^**−18**^
FST_300_mHE300	300	300	91.0%	3.7 × 10^−20^
FST_150_mHE450	150	450	90.0%	1.7 × 10^−23^
FST_0_mHE600	0	600	89.2%	5.8 × 10^−24^

*Note*: Each panel contained different ratios of high *F*_ST_ SNPs and high heterozygosity (*H*_O_) microhaplotypes. The bolded panel was chosen for further optimization. More detailed figures of GSI and kinship are available in Appendix S1.

### 3.2 Panel‐marker diversity

Following marker quality filtration, panel design, and multiplex optimization the final GTSeq panel contained 500 markers containing a total of 796 SNPs that could be grouped into 197 microhaplotype loci, which each contained more than one SNP and 303 SNP loci. The Shannon diversity of panel amplification changed from 0.28 after the first major round of optimization to 0.88 for the final panel, indicating a large increase in the evenness of sequencing while still maintaining a high level of marker richness. 269 loci aligned to unique contigs in the draft genome, and the remaining 231 loci aligned to 109 different contigs averaging 2.1 loci per‐contig and an average distance between loci aligned to the same contig of 375,627 bp. Average pairwise linkage disequilibrium (pairwise *r* ^2^) among SNPs on different loci was 0.09; however, 25% of SNPs assessed contained *r* ^2^ > 0.3 with at least one other SNP in the dataset. Microhaplotypes contained an average of 4.5 alleles (95% CI = 4.3 to 4.7), average effective number of alleles of 1.9 (95% CI = 1.8 to 2.0), and average observed heterozygosity of 0.45 (95% CI = 0.44 to 0.48). All SNPs contained two alleles, average effective number of alleles of 1.5 (95% CI = 1.48 to 1.53), and average observed heterozygosity of 0.33 (95% CI = 0.32 to 0.34). Additional breakdown of marker and population‐specific diversity can be found in Tables S5 and S6. When looking at all 500 markers, the overall *F*_ST_ of markers among collections was 0.126 (95% CI = 0.122 to 0.130) and the observed heterozygosity was 0.38 (95% CI = 0.37 to 0.39). Markers contained an average of 2.98 alleles (95% CI = 2.85 to 3.13; min = 2; max = 10) with an effective number of alleles of 1.67 (95% CI = 1.63 to 1.71). Markers had similar numbers of alleles, heterozygosity, and *G*_IS_ among collections (Table 1). The *G*_IS_ was close to zero in all collections (overall *G*_IS_ = −0.008; 95% CI = −0.013 to −0.004), and a maximum of 10% of loci departed significantly from HWE at any given collection (α = .05). No loci were significantly out of HWE once a Bonferroni correction was applied (α = .0001).

### 3.3 Among‐lake genetic stock identification

Average pairwise *F*_ST_ among Great Lakes was 0.083, the smallest distance was between Lake St. Clair and Lake Erie (*F*_ST_ = 0.008) and the largest was between Lake Erie and Lake Superior (*F*_ST_ = 0.169; Table S7). Average assignment accuracy of GSI to lake was greater than 95% for Lake Ontario (100%), Lake Erie (99%), Lake Michigan (97%), and Lake Superior (95%). Average assignment accuracy was less than 95% for Lake Huron (76%) with misassignments of individuals to Lake Michigan (9.8%), Lake St. Clair (6.4%), Lake Superior (3.5%), and Lake Erie (1.7%). Average assignment accuracy was the lowest for the Clinton River in Lake St. Clair (10%) with misassignments of individuals to Lake Erie (68%), Lake Michigan (10%), and Lake Huron (6.5%).

### 3.4 Within‐lake genetic stock identification

**FIGURE 2:**
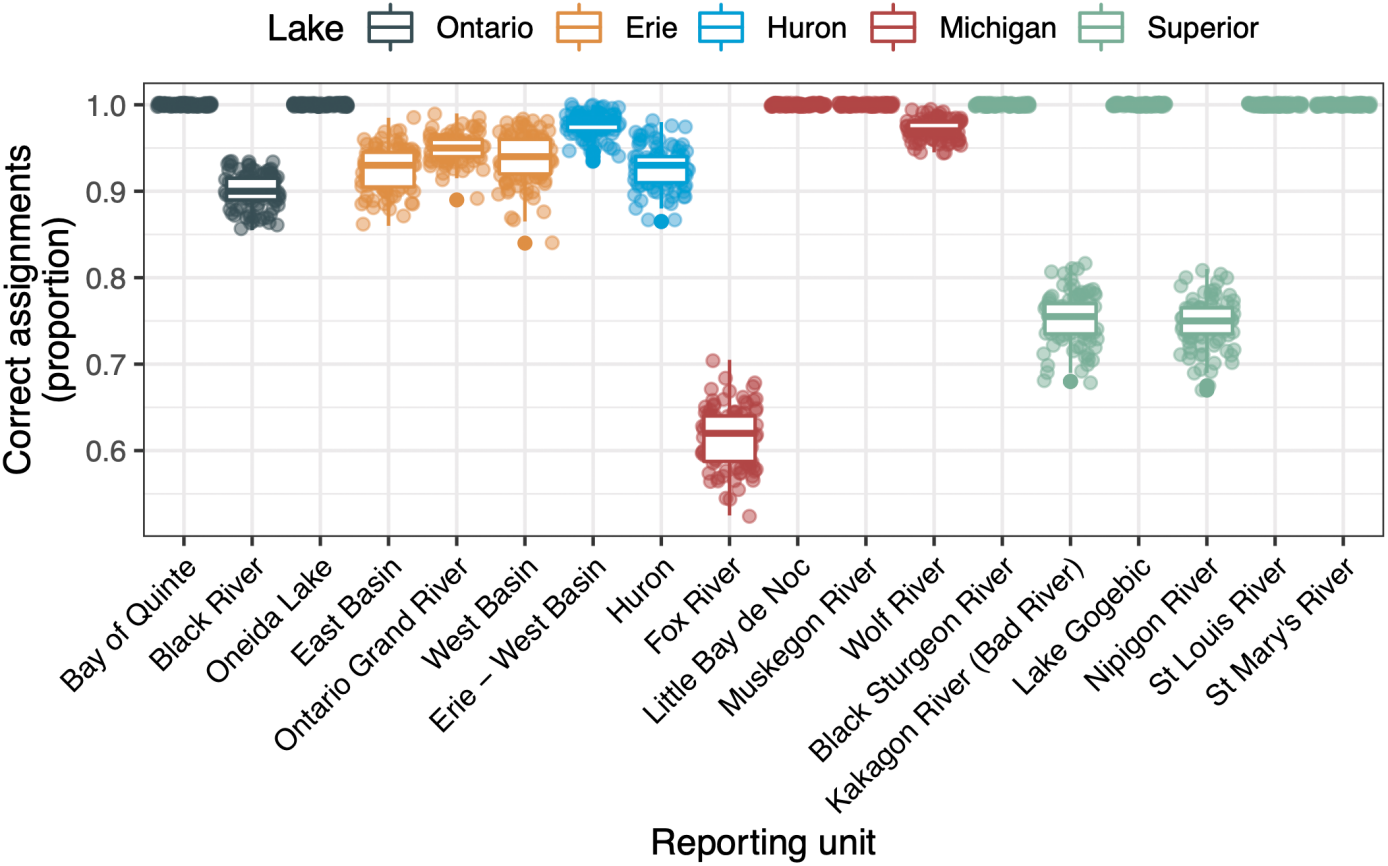
The estimated genetic stock identification accuracy for each within‐Lake reporting unit (*x‐*axis) for the final GTSeq panel containing 500 SNP and microhaplotype markers. Reporting units are colored according to their corresponding Great Lake. Each point represents the proportion of individuals correctly assigned with a (*pofZ*) score of >0.7 to a given reporting unit in a single leave‐one‐out 100% mixture simulation (*N* = 99).

To ensure that the final GTSeq panel could be used effectively within smaller jurisdictions throughout the Great Lakes, we estimated local GSI using mixture analysis and kinship within each of the Great Lakes. Within‐lake *F*_ST_ was 0.083 when averaged across all reporting group pairwise comparisons (Table S7). Among‐group pairwise *F*_ST_ was lowest among Lake Erie (average *F*_ST_ = 0.049) and highest in Lake Michigan (average *F*_ST_ = 0.097). Greater than 98.7% of individuals were assigned to at least one collection with a *pofZ* > 0.7. Of individuals with a *pofZ* score > 0.7, 80% were correctly assigned to their true collection location (Figure 2). Fox River in the Lake Michigan basin had particularly low GSI accuracy (mean = 54%). This was largely due to 35% of individuals being misassigned to the Wolf River, which is connected to the Fox River through Lake Winnebago (pairwise *F*_ST_ = 0.011). The Kakagon River and Nipigon Bay in the Lake Superior basin also had noticeably lower GSI accuracy than other collections (mean = 75%; and 77%). Walleye from the Kakagon River was primarily misassigned to the nearby St. Louis River (pairwise *F*_ST_ = 0.022), while Nipigon River fish were misassigned to multiple sites including the Kakagon River (7%; pairwise *F*_ST_ = 0.066), St. Louis River (7%; pairwise *F*_ST_ = 0.068), and St. Marys River (9%; pairwise *F*_ST_ = 0.091). Average assignment accuracy at other collections was higher than 90% but did vary among consecutive leave‐one‐out simulations.

### 3.5 Within‐lake kinship assignment

**FIGURE 3:**
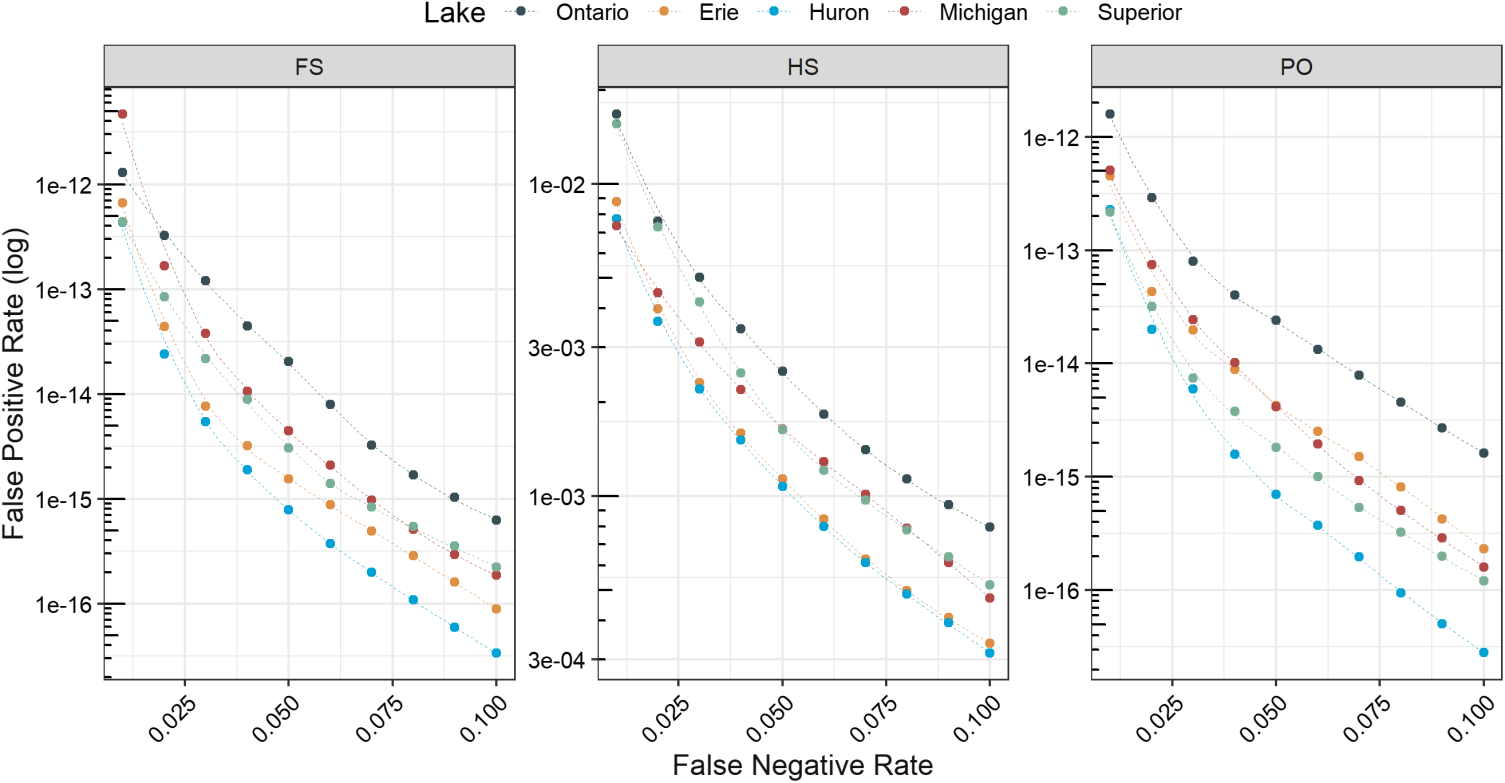
The change in false‐positive detection rates (i.e., the rate of true‐unrelated pairs being identified as full‐sibling [FS], half‐sibling [HS], or parent‐offspring [PO] pairs) for 10 false‐negative rates (0.01–0.1; i.e, the rate of true full‐sibling, half‐sibling, or parent‐offspring pairs being identified as unrelated pairs) estimated separately for each lake. Note that the *y*‐axis differs between plots.

To evaluate how well the GTSeq panel performed for kinship analysis, we compared estimates of false‐positive pairwise relationship assignments for full‐sibling, parent‐offspring, and half‐sibling relationships simulated from allele frequency distributions within each lake. False‐positive rates for full‐sibling and parent‐offspring relationships were less than 1 × 10^−11^ at an acceptable false‐negative rate of 0.01 (Figure 3). This indicates that the ability to distinguish between unrelated pairs and full‐sibling or parent‐offspring pairs was high. False‐positive rates differed slightly among lakes and were highest in lakes Erie and Huron, and lowest in Lake Ontario. However, in all cases, we concluded that the maximum false‐positive rate for full‐sibling and parent‐offspring pairs should be sufficiently low for most applications. The false‐positive rate for distinguishing true half‐siblings from unrelated pairs was substantially higher and ranged from 1 × 10^−2^ to 3 × 10^−2^ (FNR = 0.01) to 6 × 10^−4^ to 6 × 10^−4^ (FNR = 0.1). About 1 out of every 100 to 300 observations can be expected to be false positives when an FNR threshold of 0.01 is used.

### 3.6 Variation among laboratories

A similar *primer: probe* read coverage was achieved by the GLSC and UWM laboratories (UWM = 66.9X; GLSC = 77.5X). This was substantially higher than the *primer: probe* read coverage achieved by the OMNRF laboratory (OMNRF = 16.9X). Sequencing data produced by OMNRF contained a much higher number of off‐target reads per individual (average off‐target reads per individual: OMNRF = 35,593.2) compared with UWM or GLSC (UWM = 4981.6; GLSC = 6965.8).

The *primer: probe* proportion of each marker was positively correlated among runs from different laboratories suggesting that marker amplification and sequencing performed similarly between sequencing replicates (Figure S7). The correlation was weaker between OMNRF and UWM or GLSC (*r*(434) = .73, *p* < .001 and *r*(434) = .72, *p* < .001) than between GLSC and UWM (*r*(468) = .89, *p* < .001). Amplification and sequencing performance of individuals was less consistent among laboratories than markers (Figure S8). When *primer: probe* proportion was summarized by the individual, there was little correlation among sequencing runs (OMNRF to UWM *r*(94) = −.10, *p* = .316; OMNRF to GLSC *r*(94) = .42, *p* < .001; UWM to GLSC *r*(94) = .11, *p* = .297). While individual *primer: probe* proportion was not strongly correlated among runs, the variance (standard deviation [SD]) in *primer: probe* proportion among individuals within the same sequencing run was lower (SD_UWM_ = 0.12, SD_OMNRF_ = 0.03, SD_GLSC_ = 0.10) than among markers (SD_UWM_ = 0.20, SD_OMNRF_ = 0.19, SD_GLSC_ = 0.19).

**FIGURE 4:**
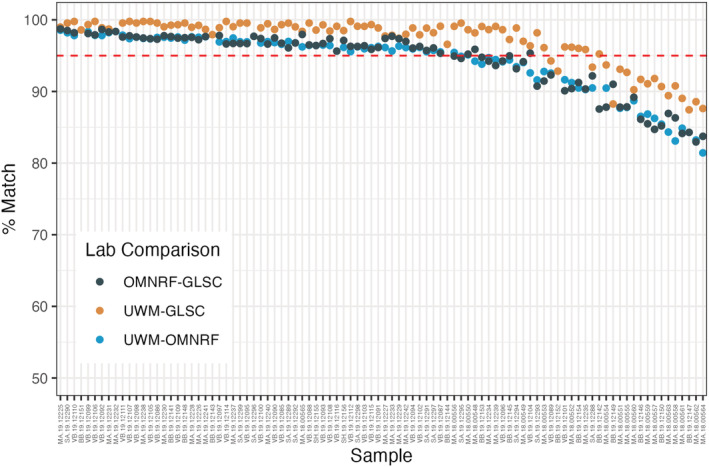
Pairwise percent of identical genotype calls (*y*‐axis) made by individual laboratories (*x*‐axis) between sequencing runs conducted independently at the Ontario Ministry of Northern Development, Mines, Natural Resources and Forestry (OMNRF) laboratory at Trent University, the University of Wisconsin‐Milwaukee (UWM) and the USGS Great Lakes Science Center, Ann Arbor MI (GLSC). The dashed red line denotes 95% congruence. Individuals are ordered approximately by percent matching calls from highest to lowest.

Individuals were successfully genotyped for 90% of markers at UWM (SD = 10.5%) and GLSC (SD = 6.3%) and 63% at OMNRF (SD = 9.9%). The average individual congruence between shared genotype calls among laboratories ranged from a low of 94% between UWM and OMNRF to a high of 97% between UWM and GLSC (Table 3). Congruence was slightly better for SNPs (95% to 98%) than microhaplotypes (93% to 96%). The range of individual congruence was large (76% to 99.8%). Average individual congruence differed among laboratories (ANOVA *p* = 6.8 × 10^−7^, *F*_2, 262_ = 1.3) with individuals from OMNRF tending to have lower congruence with UWM and GLSC than GLSC and UWM had with each other (Figure 4). Genotype congruence was not influenced by individual coverage (ANOVA *p* = .7; *F*_1, 263_ = 0.14), suggesting that depth of coverage may not be a principal factor influencing genotype congruence.

**TABLE 3:** Among‐laboratory genotype congruence statistics for individuals with a genotype rate greater than 50% for all types of markers (All), microhaplotypes (mhaps), and single nucleotide polymorphisms (SNPs).

Comparison	Median			Mean (SD)			Minimum–maximum		
	All	Mhaps	SNPs	All	Mhaps	SNPs	All	Mhaps	SNPs
UWM vs. GLSC	98.6	97.8	99.1	96.8 (4.0)	96.3 (3.8)	97.7 (3.1)	76.0–99.8	85.0–100.0	88.0–100.0
UWM vs. OMNRF	95.9	94.3	96.8	93.9 (4.3)	93.2 (4.3)	94.9 (4.4)	81.4–98.6	78.5–99.0	81.5–99.6
OMNRF vs. GLSC	96.1	94.9	97.0	94.2 (4.4)	93.2 (4.8)	95.2 (4.3)	83.0–98.7	75.0–99.2	83.3–99.6

*Note*: Values are calculated from the percentage of identical called genotypes compared between the same individuals sequenced three separate times at the University of Wisconsin—Milwaukee (UWM), the Great Lakes Science Center (GLSC), and Ontario Ministry of Natural Resources and Forestry (OMNRF). Standard deviation (SD) of the mean is shown in parentheses.

## 4 DISCUSSION

Interjurisdictional natural resource research and conservation rely on an ability to integrate data and de‐centralize work pursuing research objectives. The genotyping‐in‐thousands sequencing (GTSeq) panel that we created for walleye provides an efficient and consistent method of collecting genetic data on walleye of Great Lakes lineages for fisheries research and management purposes. We demonstrate that SNP and microhaplotype genotypes from the 500 markers included in our GTSeq panel could be used to: (1) assign individuals to most major walleye stocks in each of the Great Lakes with >90% accuracy; (2) assign parent‐offspring, full‐sibling, and half‐sibling kinship relationships with low false‐positive rates of detection; and (3) reproduce genotypes in separate sequencing runs on different sequencers at different facilities on average > 94% of the time.

### 4.1 Predicted performance for fisheries applications

Identification of the geographical source of a sample of unknown origin has important implications for both management (Valenzuela‐Quiñonez, 2016) and conservation biology (Zhang et al., 2020). By targeting genetic markers with high diversity and among‐collection allele frequency variability, we created a multi‐use GTSeq panel that should perform adequately for most walleye GSI studies in major Great Lakes jurisdictions. Stock identification and structure is a key management objective for several major walleye population assemblages throughout the Great Lakes including Lake Erie (Euclide, MacDougall, et al., 2021), Saginaw Bay, Lake Huron (Brenden et al., 2015), Green Bay, Lake Michigan (Dembkowski et al., 2018), and Lake Superior (Homola, *unpublished data*). Our analysis shows that the panel should perform sufficiently well in each of these regions to assign individuals to specific spawning reefs/sites as in Lake Superior or to groups of sites such as the “West Basin” vs. “East Basin” of Lake Erie with >90% accuracy. Importantly, this means that this single marker panel could be used to facilitate mixed‐stock assignment and recovery programs for walleye in many different areas. Data collected from these regional studies could be shared to identify long‐distance migrants and larger spatial patterns in movement and gene flow.

Future generation and sharing of new data by researchers using this GTSeq panel could help to improve GSI and kinship assignment accuracy. Increasing the number of sites and samples included in population baselines increases the accuracy of population allele frequency estimates (Wood et al., 1987). In our study, sample sizes of our baseline dataset were variable but generally included greater than 30 individuals from a given spawning population. The high GSI and kinship accuracy at the lake and collection levels suggest that our samples provided an adequate baseline for common management applications. However, additional sampling and genotyping from new sites and new individuals from collections with low sample sizes (*N* < 30) would improve allele frequency estimates, especially for microhaplotype data. The importance of sample size is exemplified by the GSI accuracy in Lake Erie, where the pairwise *F*_ST_ among reporting groups is low compared with the rest of the Great Lakes, but GSI accuracy was still greater than 90%, which we attribute to the large sample sizes available for those reporting groups. However, increasing the baseline dataset through data sharing must be done with caution. Our results indicate that genotype congruency was not 100% among separate sequencing runs. Therefore, the use of reference samples and continued assessments of GTSeq panel genotype accuracy would be necessary to ensure that there is consistency in genotype scoring between the existing baseline and newly added samples. Extensive baseline genotyping and development of allele frequency reference samples are important steps in the development of standardized marker panels (Seeb et al., 2007; Stott et al., 2010). Therefore, the present panel should be viewed as the starting place that will be improved with ongoing collaboration and continued optimization.

One of the major benefits of including microhaplotype loci in panel construction is that they provide multiallelic markers that can facilitate kinship and pedigree analysis (Baetscher et al., 2018). Our data demonstrated that microhaplotypes did contain higher genetic diversity than biallelic SNPs, which contributed to accurate kinship assignment for walleye throughout the Great Lakes. However, microhaplotypes also contained higher inter‐laboratory scoring errors. These data could provide new opportunities to assess the abundance of local walleye populations using genetic techniques such as close‐kin mark‐recapture (CKMR) and rarefaction, which benefit from multiallelic markers (Bravington et al., 2016; White et al., 2022).

Prior to the application of the present panel to kinship studies, there are several reasons why additional assessments of kinship for target populations will be necessary. First, the false‐positive rates of detection for half‐siblings were substantially higher than for parent‐offspring and full‐sibling identification in simulations. Misassignment of half‐siblings can be an issue for CKMR when full‐sibling and parent‐offspring pairs may be uncommonly encountered in sample sets (Waples & Feutry, 2022). Second, our analysis focused on determining false‐positive rates of misassigning an unrelated pair as a related pair. However, the majority misassignments are likely to occur between different types of related pairs (e.g., misassigning half‐siblings as full‐siblings). Third, about a quarter of the SNP markers in the panel appear to be in moderate linkage disequilibrium with at least one other locus in the panel. Given the large physical distance between markers based on alignment to the draft walleye genome, we suggest that much of this linkage is the result of population structure and not physical linkage among loci. Nonetheless, power assessments of kinship assignment can become inflated when linked loci are included (Huang et al., 2004). Thus, researchers should conduct their own power assessments and linkage disequilibrium assessments using samples collected from their study area to determine the statistical power of the panel prior to large‐scale application.

### 4.2 Interjurisdictional collaboration

Most fisheries management and research activities in the Great Lakes are decentralized and decisions are based on data produced from each lake's surrounding jurisdictional fisheries agencies. Therefore, the creation of a standardized resource is only the first step towards unifying walleye research and stock monitoring throughout the Great Lakes region (Sard et al., 2020; Stott et al., 2010). Long‐term collaboration among laboratories will be required to ensure that data produced separately is consistent and comparable. We demonstrated that most genotype calls were consistent among independent sequencing runs; however, discrepancies can be expected. For example, sequencing data produced from OMNRF contained fewer reads that could be assigned to any of the target markers, and this led to a lower overall genotyping rate for individuals in this dataset. We were unable to identify the reason for the lower sequencing quality obtained from the OMNRF laboratory; however, we predict that it is likely associated with slight differences in laboratory protocols. While OMNRF data were generated using Nextera XT adapter instead of the Small RNA Primer used at UWM and the GLSC, we believe it is unlikely that this led to major differences in sequencing quality as the Nextera XT adapter is compatible with the Illumina MiSeq technology (Illumina, San Diego, CA, USA). Several individuals in our dataset showed consistently lower congruency, but we did not find any clear relationship with reading counts or *primer: probe*, suggesting that other factors may influence individual congruency. Further publications of GTSeq genotype error rates and the establishment of a reference sample database may help to increase consistency among laboratories. Similar approaches have been successful for microsatellite panels (Seeb et al., 2007; Stott et al., 2010) and have begun to be used for GTSeq panels (Bohling et al., 2021; Hayward et al., 2022). However, the appropriate use of positive and negative controls should help account for batch effects in future studies.

The need for standardized resources that facilitate interjurisdictional research is a constant across natural resource conservation and management. Here we respond to that need by developing a new genetic resource that will facilitate population structure and connectivity research of one of the most important fisheries in the Great Lakes region of the United States and Canada, walleye. Our panels and necessary resources have been made publicly available through this publication (Dryad: https://doi.org/10.5061/dryad.xd2547dmg). We showed that the GTSeq panel provides high assignment accuracy for major walleye stocks in each of the Great Lakes, low false‐positive kinship assignment for full‐sibling and parent‐offspring pairs, and >95% genotype congruence among subsequent sequencing runs. We hope that future studies using this research will continue to improve panel performance and add to ongoing collaboration to the benefit of walleye fisheries in North America.

## AUTHOR CONTRIBUTIONS

**Wesley A. Larson:** Conceptualization (lead); data curation (supporting); formal analysis (supporting); funding acquisition (lead); investigation (equal); methodology (equal); project administration (equal); resources (equal); supervision (equal); validation (equal); visualization (supporting); writing – original draft (supporting); writing – review and editing (supporting). **Matthew Bootsma:** Formal analysis (supporting); methodology (supporting); visualization (supporting); writing – original draft (supporting). **Loren Miller:** Conceptualization (equal); funding acquisition (equal); writing – original draft (supporting); writing – review and editing (supporting). **Kim T. Scribner:** Conceptualization (equal); funding acquisition (equal); writing – original draft (supporting); writing – review and editing (supporting). **Wendylee Stott:** Conceptualization (equal); funding acquisition (equal); investigation (supporting); methodology (supporting); resources (supporting); writing – review and editing (supporting). **Chris C. Wilson:** Conceptualization (equal); formal analysis (supporting); funding acquisition (equal); investigation (supporting); methodology (supporting); project administration (supporting); writing – original draft (supporting); writing – review and editing (supporting). **Emily K. Latch:** Funding acquisition (supporting); investigation (supporting); project administration (supporting); supervision (equal); writing – original draft (supporting); writing – review and editing (supporting). **Peter T. Euclide:** Conceptualization (lead); data curation (lead); formal analysis (lead); funding acquisition (supporting); investigation (lead); methodology (lead); project administration (equal); visualization (lead); writing – original draft (lead); writing – review and editing (lead).

### OPEN RESEARCH BADGES

This article has earned Open Data and Open Materials badges. Data and materials are available at https://doi.org/10.5061/dryad.xd2547dmg.

## BENEFIT‐SHARING STATEMENT

An international research collaboration was developed with scientists from the nations and states providing genetic samples, and many of those collaborators have been included as co‐authors. The results of this study are being shared openly with all agencies involved in walleye management and made accessible to the broader scientific community through this publication. Our group is committed to scientific partnerships and to developing a more inclusive and open space for research.

## Supporting information


Appendix S1

Figures S1–S8

Tables S1–S8

## Data Availability

Datasets, including primer sequences, capture bait fasta file, GTScore input files, and all of the sample metadata and GTSeq genotyping data used to generate the figures and tables included in the main body of the text, will be made available on Dryad upon manuscript acceptance (Euclide et al., 2022).
