# Supplementary material for: A new GTSeq resource to facilitate multijurisdictional research and management of walleye Sander vitreus
Source: Ecol Evol. 2022 Dec 14;12(12):e9591. doi: 10.1002/ece3.9591 (PMC9750844; doi:10.1002/ece3.9591)
Supplement: Supplementary file 1 — Appendix S1 [file ECE3-12-e9591-s002.docx]

**File S1: Supplementary methods used in panel development**

*Initial RAD Sequencing*

Single nucleotide polymorphisms (SNPs) were identified by conducting *PSTI* RAD-sequencing (Ali et al. 2016) of 45 walleye. Fin clips from eight to ten walleye from each lake were sent to the Molecular Conservation Genetics Laboratory at the University of Wisconsin Stevens Point where DNA was extracted using a Qiagen DNEasy Blood and Tissue Kit and standard extraction protocols. Samples were then prepared for sequencing following the BestRAD protocol (Ali et al. 2016; Ackiss et al. 2020) using the *PSTI* enzyme for restriction enzyme digestion. The pooled library was then sequenced at Novogene (Sacramento, CA) on a single Novaseq S4 lane. The DNA sequence data was processed using the STACKS v.2.3 *de novo* pipeline (Rochette et al. 2019; Table S8). First, samples were demultiplexed using *process_radtags* which removes low quality and un-barcoded reads and assigns reads to individual samples. Following *process_radtags,* a total of 3,530,996,406 reads were retained and assigned to individuals (mean reads per individual = 78 million). Stacks of highly similar sequences were generated for each individual in *ustacks*. Next, the four individuals from each lake with the highest number of retained reads were used to generate a catalog of putative loci using *cstacks* and individual reads of all samples were matched to the catalog via *sstacks*. Finally, individual sequence matches were converted to bam format (*tsv2bam*) and SNPs were identified at each locus and individuals were genotyped using *gstacks* and the results were output in VCF format using *populations*.

*RADCapture design*

Initial RAD sequencing identified 1,561,529 putative SNPs with a minor allele count greater than 3. To ensure that loci included in the capture panel were not the product of sequencing errors or otherwise compromised, SNPs were filtered for quality control using VCFTools version 0.1.16 (Table S1). First, individuals with high degrees of missing data were removed, this included four individuals from Lake Ontario with > 90% missing data. Next, SNPs with extremely high degrees of missing data were removed, therefore, SNPs that were genotyped in less than 20% of individuals were removed from the dataset. Finally, to eliminate physical linkage among SNPs aligned to the same RAD-tags, we removed all but the SNP with the highest minor-allele frequency (MAF) from every RAD-tag. Finally, to reduce the likelihood that SNPs were the result of sequencing or alignment error, any locus with a MAF < 0.05 was removed. These filters combined resulted in a final dataset of 505,225 loci. To maximize genome coverage, we aligned RAD-tags to an early draft walleye genome and selected panel loci based on alignment position. All 505,225 RAD-tags were aligned using bowtie2 version 2.2.4 and the –fast default settings (Langmead and Salzberg 2012). A total of 419,018 RAD-tags had high quality alignments based on their mapping quality score. This score is an estimate of sequence alignment uniqueness that ranges from 0 (poor) to 42 (high). All alignments with a quality score less than 10 were removed. Next, sequence alignments were filtered using a custom R script to remove SNPS found on contigs < 0.1 MB long or that were within 5 kb of an adjacent SNP. This resulted in a total of 129,281 loci spaced approximately evenly across the largest contigs. To obtain a final panel of 100k loci for bait development, loci were filtered again based on MAF by selecting the 100k loci with the highest MAF which resulted in 100,492 loci from which 492 loci with the lowest MAF were randomly removed. The final panel sent for bait creation contained exactly 100k loci and had an average MAF of 0.292 (min=0.144, max=0.5) and average expected heterozygosity of 0.386 (min=0.202, max = 0.5).

Sequence data for all 100,000 loci were sent to Arbor Bioscience (Ann Arbor, MI) who designed a single 80 nucleotide bait at 2X tiling density for each of the 100,000 sequences containing a SNP of interest. Baits were BLASTed against the yellow perch genome (Feron et al. 2020) keeping only baits that passes “Stringent” BLAST filtering, matched a region of the genome that was <= 25% soft masked of repeats, and did not align to the mitochondrial genome. The bait closest to the 5’ end of each sequence was retained. Only 364 loci did not meet QC for bait design resulting in a final set of 99,636 capture baits spanning 99,636 loci in the walleye genome. As a test of panel performance prior to large scale application the Rapture panel, the population structure of the 45 walleye included in the initial RAD-Seq survey was described using principal component analysis (PCA) which suggested that lake-level population structure could be determined using the first three axes of the PCA (Figure S2).

*RADCapture sequence methods*

To comprehensively survey genetic diversity present in the Great Lakes walleye population, 1,289 walleye from 29 spawning sites were sequenced using the Rapture panel following library preparation protocols outlined in Ali et al. (2016). Individuals were sequenced using paired-end 150 sequencing on three Novaseq S4 lanes at Novogene along with 438 walleye being sequenced for another project (Sacramento, CA). Sequence data was demultiplexed and processed using STACKS 2.3 (Rochette et al. 2019; total retained reads = 11,011,232,431) using the same set of parameters and catalog that was used to analyze the initial RAD-seq data during the panel development phase with the exception of the *populations* step which used more stringent filters and included a whitelist to filter to only those loci that with capture baits (-R 0.2 –min-mac 4; Table S8). Following initial quality filtering, individual samples retained an average of 8.5 million reads spanning 78,997 out of the initial 99,636 baited loci (79%). These RAD-tags contained 340,597 putative SNPs with a minor allele count > 4 and present in at least 20 percent of the 31 sites. Sequence data was filtered for quality using a similar, but more stringent approach as initial RAD sequencing (Table S2). One hundred and nineteen individuals that were missing genotypes at greater than 90% of SNPs and were removed, and 138,895 SNPs that failed to genotype in greater than 50% of individuals were also removed. Individuals were reassessed for missing data and an additional 102 individuals were removed that were missing genotypes at greater than 50% of the remaining SNPs. To eliminate bias created by linkage among SNPs on the same sequence read, all but the SNP with the highest minor-allele frequency was removed from each locus, and then the entire dataset was filtered again to retain only SNPs that were genotyped in at least 70% of individuals. This resulted in a final retention of 1,069 individuals (83%) and 44,261 SNP loci of the 99,636 included in the Rapture panel (44%). While it is unfortunate that so many individuals and loci had to be removed during quality filtering, this loss was not unexpected. Many of the DNA samples sequenced came from older, partially degraded DNA samples that had been frozen and thawed multiple times since extraction or from dried spine samples that often produce low-concentration DNA. Therefore, whenever possible, we used large initial sample sizes for sites that contained low-quality tissue or DNA to ensure that poorly sequenced samples could be removed without greatly impacting the robustness of the dataset. As for loci, analysis of other Rapture datasets has shown that retention of baited loci is often incomplete. For example, the panel used in Euclide et al. (2021) contained two baits per-marker, which is supposed to increase bait success, and still only 70% of loci were retained following similar quality filtering. Also, Reid et al. (2021) found that retention of baited loci was between 50 and 60% for their panel which also contained multiple baits for each locus. Therefore, our results appear to be comparable to previous research suggesting that while Rapture panels do help to obtain a more consistent set of markers than RAD-seq, many markers are still lost during quality filters. A population genetic survey using the 44,261 SNP loci was conducted to calculate population diversity estimates and to identify markers to target for a GT-seq panel. Results of PCA indicated that population structure was present at both the level of individual lakes and globally across the Great Lakes (Figure S3).

*GT-seq panel marker selection*

Genetic stock identification of the five simulated original marker combinations and the final panel was assessed using Rubias (see main text; Moran and Anderson 2019). Genetic stock identification was assessed using microhaplotype data for individuals grouped by 1) collection, and 2) collections grouped into 8 reporting units based on geography. Performance was evaluated by using simulated 100% mixtures and a leave-one-out procedure following approaches outlined in the Rubias walkthrough documentation (<https://github.com/eriqande/rubias#assessment-of-genetic-references>). Each panel was evaluated using the *assess.reference.loo()* function, 600 replicates and a mixture size of 200 individuals. Correct assignment was defined as individuals with a PofZ score >0.7 for the known collection or reporting unit closely following protocols outlined in Bootsma et al. (2020, Figure S6). Relatedness was assessed following protocols outlined in Bootsma et al., (2020) and Baetscher et al. (2019) using CKMRsim (﻿Anderson, https://zenodo. org/record/820162). This approach uses log-likelihood ratios between a tested relationship and the hypothesis of no-relationship calculated from genotype-pair probabilities for related individuals simulated from observed allele frequency estimates. The distributions of log-likelihood ratios for different relationships are then used to estimate false positive and false negative rates which were used as a measure of relative panel performance.

For the purpose of panel design, walleye stocks were grouped into eight reporting units that were identified using a combination of principal component analysis (PCA) clustering and prior knowledge of the system. Diversity statistics within each of these units were used to test the performance of five different possible marker filtering parameters. Reporting units included Lake Superior stocks, the St. Mary’s River stock which connects Lake Superior to Lake Huron/Michigan, Lake Michigan stocks, Lake Hurons stocks, Lake Ontario stocks, the east and west basin on Lake Erie, and the Ontario Grand River. The three reporting units in Lake Erie were used to account for previous research that identified these regions as identifiable units for mixed-stock analysis (Euclide et al. 2021). Genetic stock identification to each of these reporting units was similar among all five panels but increased as higher numbers of *F*_ST_ SNPs were included. In all filtering scenarios, correct assignment was lowest for Lake Huron owning to the miss assignment to lakes Erie and Michigan. The Saginaw Bay/Tittabawassee River walleye population in Lake Huron collapsed the mid-1940s, and stocking began in the 1970s and 1980s using fingerlings from the Muskegon River, Lake Michigan (Fielder and Baker 2004; Brenden et al. 2015). Additionally, walleye from the west basin of Lake Erie are known to migrate to Saginaw Bay, and therefore the genetic makeup of Saginaw fish likely represents an admixture of Muskegon and Lake Erie walleye (Brenden et al., 2015). All other reporting groups showed genetic stock assignment greater than 80% for all maker filtering scenarios (Figure S4). Genetic stock identification of the mix600 panel following primer design, but prior to PCR optimization had a GSI similar to the Fst450_mHE150 from which it was modeled off. Following the loss of 100 markers during PCR optimization, GSI of the final mix500 panel was similar to the mix600 panel suggesting that very little statistical power was lost during optimization.

False positive rate of identifying full sibling and parent-offspring pairs was universally low for all five panels (Figure S5). Increasing the number of high heterozygosity microhaplotypes used in the panel decreased the false positive rate, therefore the FST0_mHE600 panel had the lowest FPR, while the chosen combination (Fst450_mHE150) had intermediate FPR (Figure S5). The mix600 panel following primer design, but prior to PCR optimization had an FPR similar to the Fst450_mHE150 panel as expected. Following the loss of 100 markers during PCR optimization some of which were diverse microhaplotype loci, the final mix500 panel had a slightly higher FPR, but in all cases FPR was low enough for panels to adequately perform relationship reconstruction.

*References*

Ackiss, A.S., Larson, W.A., and Stott, W. (2020). Genotyping-by-sequencing illuminates high levels of divergence among sympatric forms of coregonines in the Laurentian Great Lakes. Evol. Appl. **13**(5): 1037–1054. doi:10.1111/eva.12919.

Ali, O.A., O’Rourke, S.M., Amish, S.J., Meek, M.H., Luikart, G., Jeffres, C., and Miller, M.R. (2016). Rad capture (Rapture): Flexible and efficient sequence-based genotyping. Genetics **202**(2): 389–400. doi:10.1534/genetics.115.183665.

Baetscher, D.S., Anderson, E.C., Gilbert‐Horvath, E.A., Malone, D.P., Saarman, E.T., Carr, M.H., and Garza, J.C. (2019). Dispersal of a nearshore marine fish connects marine reserves and adjacent fished areas along an open coast. Mol. Ecol. **28**(7): 1611–1623. doi:10.1111/mec.15044.

Bootsma, M.L., Gruenthal, K.M., McKinney, G.J., Simmons, L., Miller, L., Sass, G.G., and Larson, W.A. (2020). A GT-seq panel for walleye (*Sander vitreus*) provides important insights for efficient development and implementation of amplicon panels in non-model organisms. Mol. Ecol. Resour. **20**(6): 1706–1722. John Wiley & Sons, Ltd. doi:10.1111/1755-0998.13226.

Brenden, T.O., Scribner, K.T., Bence, J.R., Tsehaye, I., Kanefsky, J., Vandergoot, C.S., and Fielder, D.G. (2015). Contributions of Lake Erie and Lake St. Clair walleye populations to the Saginaw Bay, Lake Huron, recreational fishery: Evidence from genetic stock identification. North Am. J. Fish. Manag. **35**(3): 567–577. doi:10.1080/02755947.2015.1020079.

Euclide, P.T., MacDougall, T., Robinson, J.M., Faust, M.D., Wilson, C.C., Chen, K.-Y.Y., Marschall, E.A., Larson, W., and Ludsin, S. (2021). Mixed-stock analysis using Rapture genotyping to evaluate stock-specific exploitation of a walleye population despite weak genetic structure. Evol. Appl. **14**(5): 1403–1420. Cold Spring Harbor Laboratory. doi:10.1111/eva.13209.

Feron, R., Zahm, M., Cabau, C., Klopp, C., Roques, C., Bouchez, O., Eché, C., Valière, S., Donnadieu, C., Haffray, P., Bestin, A., Morvezen, R., Acloque, H., Euclide, P.T., Wen, M., Jouano, E., Schartl, M., Postlethwait, J.H., Schraidt, C., Christie, M.R., Larson, W.A., Herpin, A., and Guiguen, Y. (2020). Characterization of a Y‐specific duplication/insertion of the anti‐Mullerian hormone type II receptor gene based on a chromosome‐scale genome assembly of yellow perch, Perca flavescens. Mol. Ecol. Resour. **20**(2): 531–543. doi:10.1111/1755-0998.13133.

Fielder, D.G., and Baker, J.P. (2004). Strategy and options for Completing the Recovery of Walleye in Saginaw Bay, Lake Huron. *In* STATE OF MICHIGAN DEPARTMENT OF NATURAL RESOURCES: FISHERIES DIVISION, SPECIAL REPORT. Lansing, MI. Available from https://www.researchgate.net/publication/298810500_Strategy_and_options_for_completing_the_recovery_of_walleye_in_Saginaw_Bay_Lake_Huron_David_G_Fielder_and_James_P_Baker [accessed 9 May 2022].

Langmead, B., and Salzberg, S.L. (2012). Fast gapped-read alignment with Bowtie 2. Nat. Methods **9**(4): 357–359. doi:10.1038/nmeth.1923.

Moran, B.M., and Anderson, E.C. (2019). Bayesian inference from the conditional genetic stock identification model. Can. J. Fish. Aquat. Sci. **76**(4): 551–560. Canadian Science Publishing. doi:10.1139/cjfas-2018-0016.

Reid, B.N., Moran, R.L., Kopack, C.J., and Fitzpatrick, S.W. (2021). Rapture-ready darters: Choice of reference genome and genotyping method (whole-genome or sequence capture) influence population genomic inference in Etheostoma. Mol. Ecol. Resour. **21**(2): 404–420. Mol Ecol Resour. doi:10.1111/1755-0998.13275.

Rochette, N.C., Rivera-Colón, A.G., and Catchen, J.M. (2019). Stacks 2: Analytical methods for paired-end sequencing improve RADseq-based population genomics. Mol. Ecol. **28**(21): 4737–4754. doi:10.1111/mec.15253.
