# Supplementary material for: A new GTSeq resource to facilitate multijurisdictional research and management of walleye Sander vitreus
Source: Ecol Evol. 2022 Dec 14;12(12):e9591. doi: 10.1002/ece3.9591 (PMC9750844; doi:10.1002/ece3.9591)
Supplement: Supplementary file 2 — Figures S1–S8 [file ECE3-12-e9591-s003.docx]

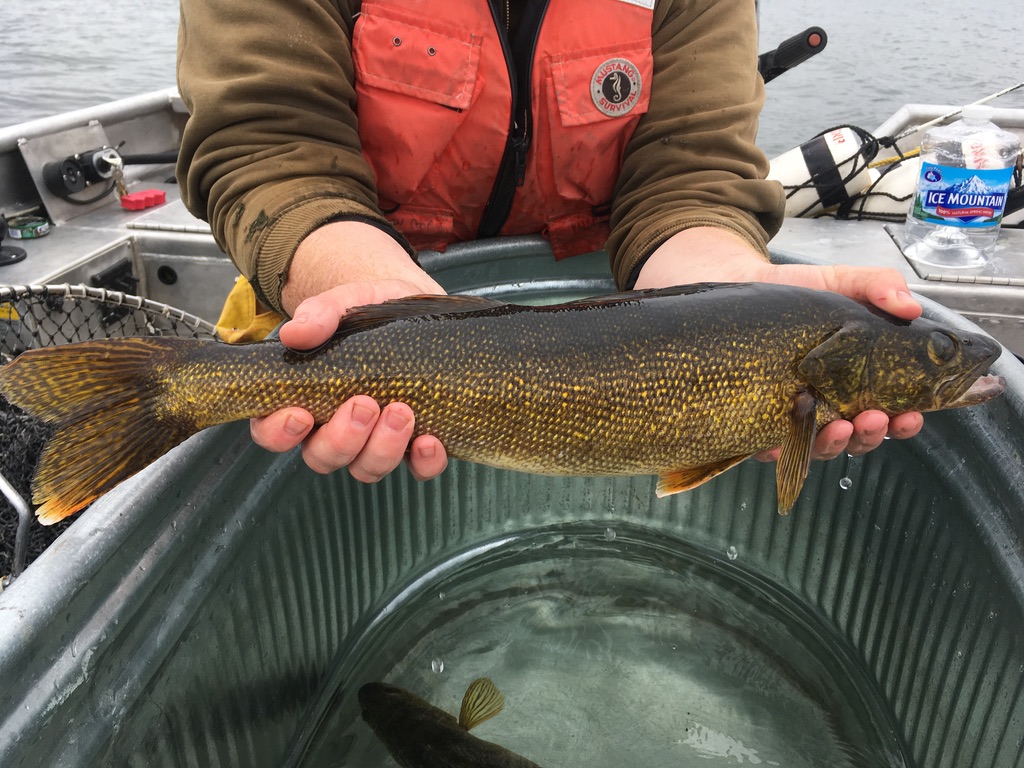


Fig. S1: Image of walleye (*Sander vitreus*) collected from Green Bay, Lake Michigan. Photo taken by Danial Isermann, University of Wisconsin Stevens Point


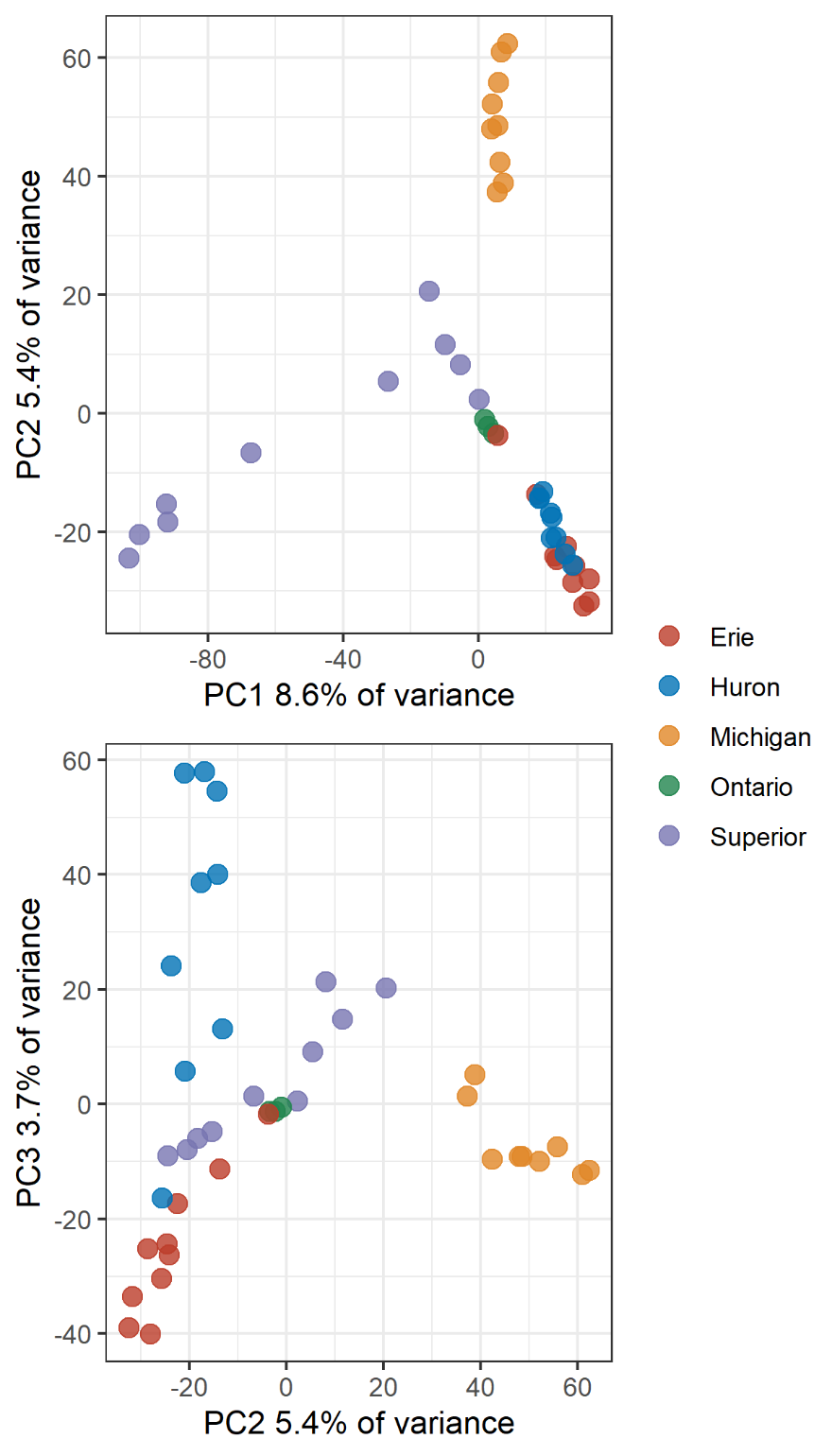


Fig. S2: Principal component analysis comparing 45 walleye sampled from lakes Erie, Huron, Michigan, Ontario, and Superior based on the 100,000 SNP loci submitted to ArborBioscience for capture bait design.


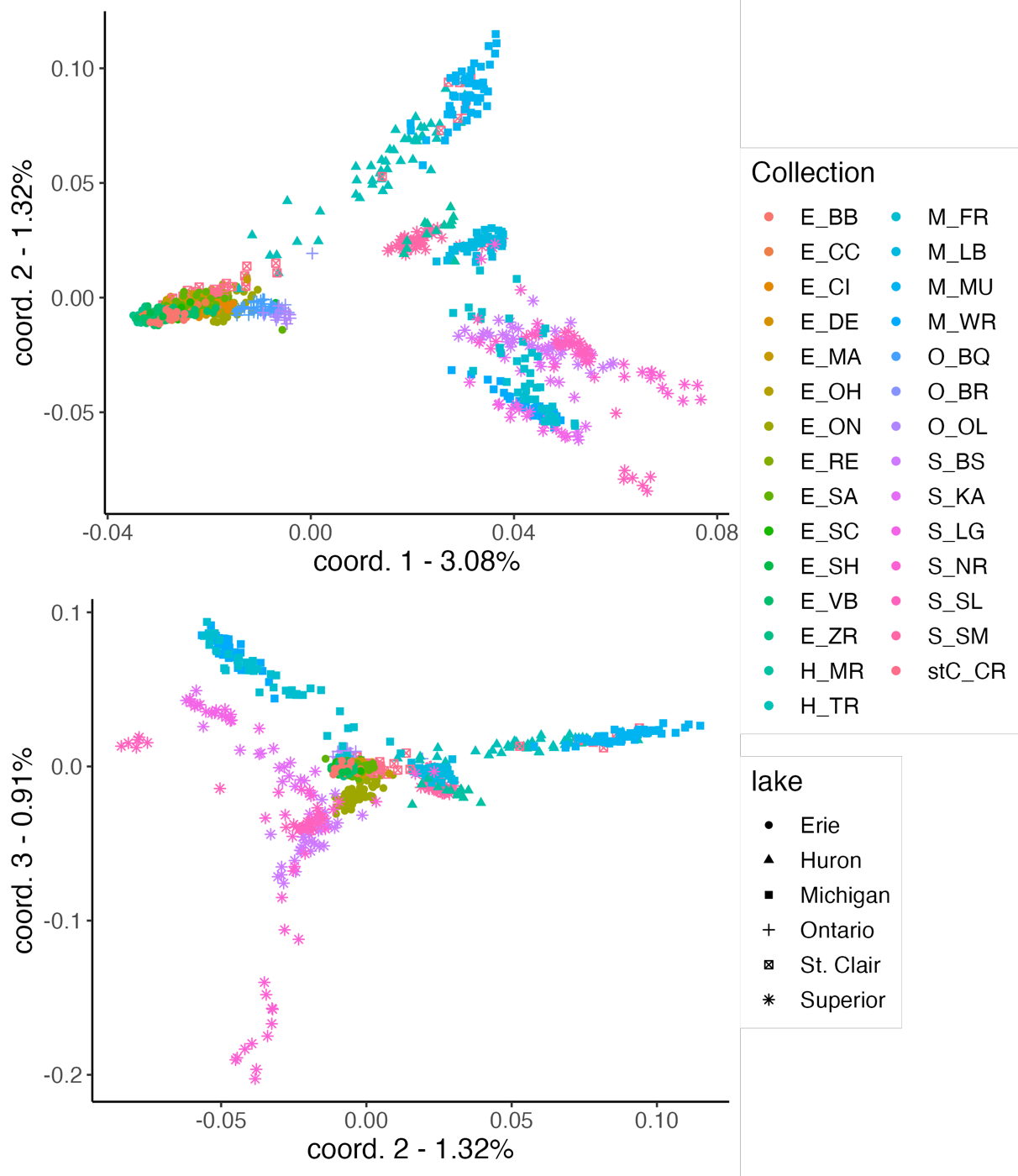


Fig. S3: The first three principal components of a PCA comparing 1,289 walleye sampled from lakes Erie, Huron, Michigan, Ontario, and Superior using the 44,261 SNP loci used to develop the GTSeq panel. Population codes used in the Figure legend and lakes can be found in Fig S2 supporting table on the following page.

Fig. S3 supporting table: Sample collections and number used for Rapture population genetic assessment. Site codes correspond to legends in the following principal component analysis (PCA) bi-plots.

| Lake | Population | Population Code |
| --- | --- | --- |
| Erie | Bournes Beach | E_BB |
| Erie | Cattaragus Creek | E_CC |
| Erie | Chicken Island Reef | E_CI |
| Erie | Detroit River | E_DE |
| Erie | Maumee River | E_MA |
| Erie | Grand River, Ohio | E_OH |
| Erie | Grand River, Ontario | E_ON |
| Erie | Tourssant/Niagra Reef | E_RE |
| Erie | Sandusky River | E_SA |
| Erie | Lackawanna Shoal | E_SC |
| Erie | Shorehaven | E_SH |
| Erie | Van Buren Bay | E_VB |
| Erie | Zellerhouse Reef | E_ZR |
| Lake St. Clair | Clinton River | stC_CR |
| Huron | Moon River | H_MR |
| Huron | Tittabawassee River | H_TR |
| Michigan | Fox River | M_FR |
| Michigan | Little Bay de Noc | M_LB |
| Michigan | Muskegon | M_MU |
| Michigan | Wolf River | M_WR |
| Ontario | Bay of Quinte | O_BQ |
| Ontario | Black River | O_BR |
| Ontario | Oneida Lake | O_OL |
| Superior | Black Sturgeon Bay | S_BS |
| Superior | Kakagon River (Bad River) | S_KA |
| Superior | Lake Gogebic | S_LG |
| Superior | Nipagon Bay | S_NB |
| Superior | Nipagon River | S_NR |
| Superior | St. Louis River | S_SL |
| Superior | St. Marys River | S_SM |


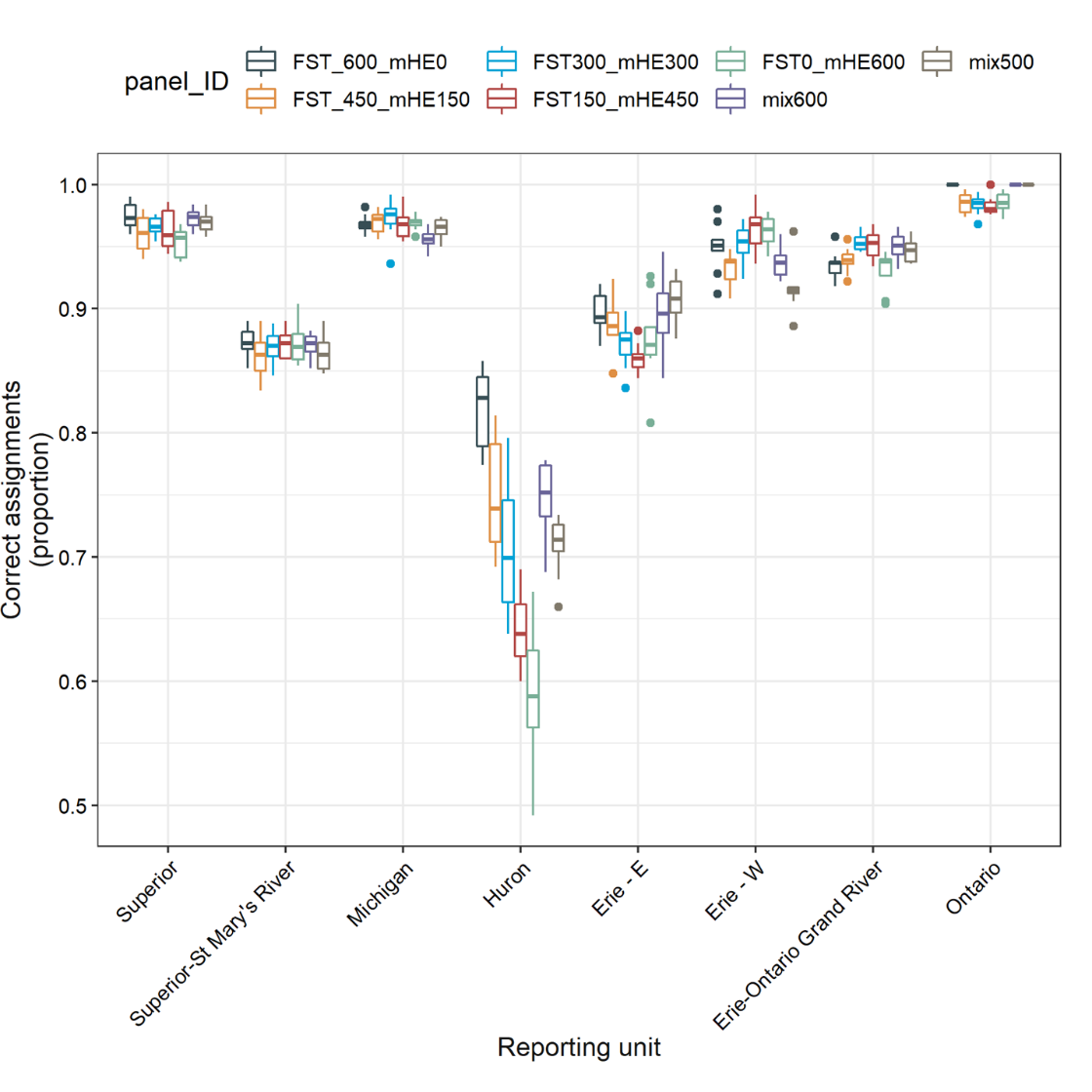


Fig S4: Estimated percent correct genetic stock identification for five tested marker selection ratio approaches, the panel of 600 markers that primers were purchased for and the final panel containing 500 markers. Reporting units (x-axis) were defined based on groupings along the first two axes of a principal component analysis and prior-knowledge of the system (i.e., existing jurisdictional and geographic breaks in the system).


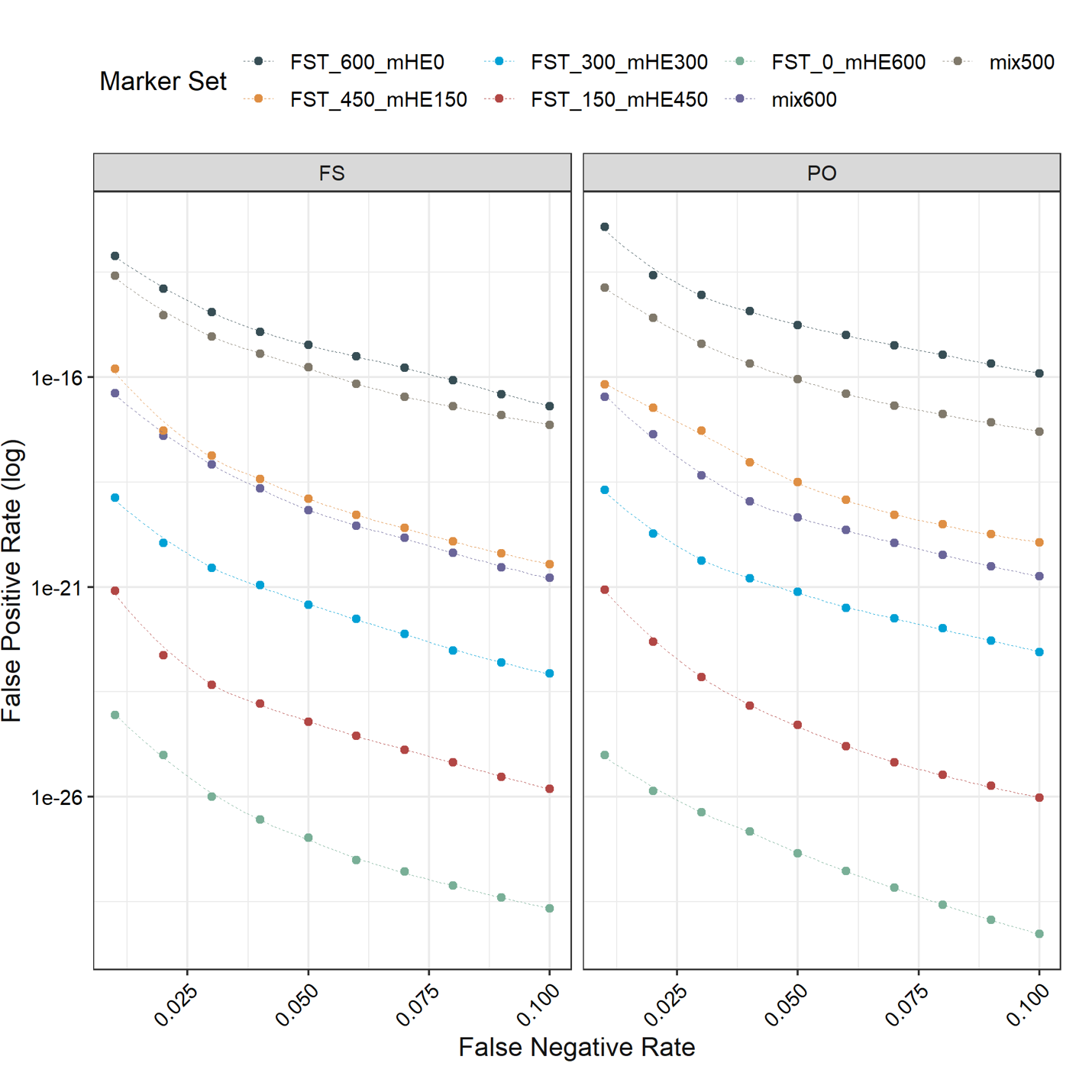


Fig. S5: Estimated false positive rate of full sibling (FS) and full parent (PO) identification for five tested marker selection ratio approaches, the panel of 600 markers that primers were purchased for and the final panel containing 500 markers. Note that the y-axis values have been log transformed.


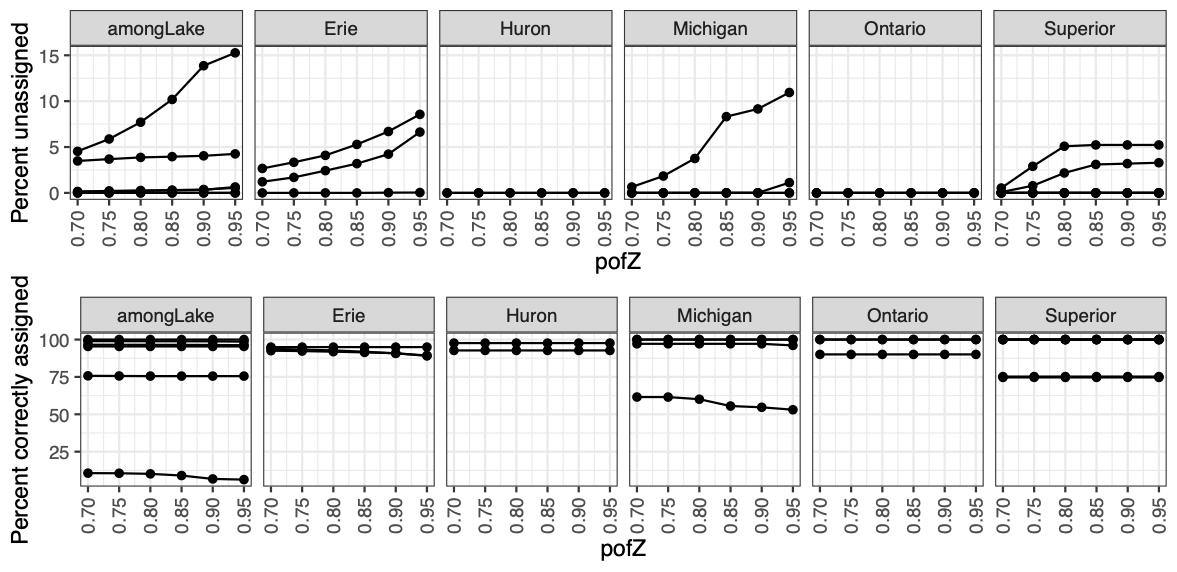


Fig. S6: Comparison of the influence that pofZ threshold of 0.7 to 0.95 in 0.05 unit increments has on the percent of individuals left unassigned (top) and percent of individuals correctly assigned (GSI Accuracy, bottom). Each line represents the score for a different collection within a tested testing region. These regions reflect the same regions used in the final panel performance analysis discussed in the manuscript.


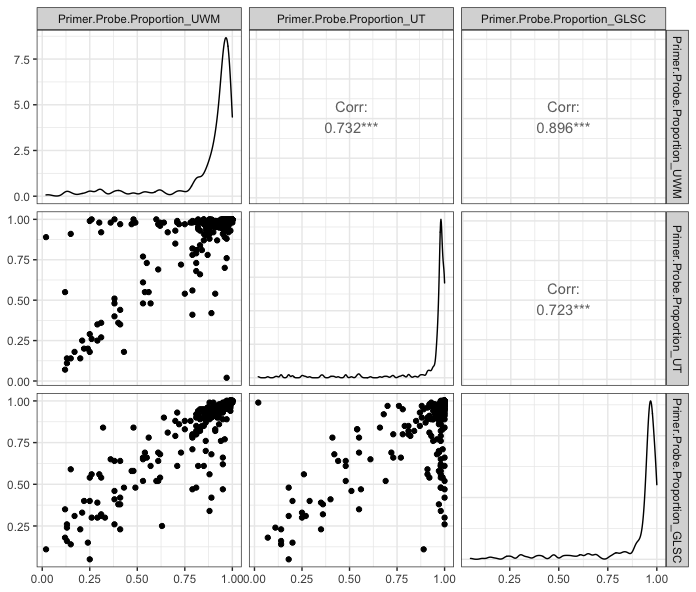


Fig. S7: Pairs-plot comparing *primer:probe* proportion identified for all 500 markers in the GT-seq panel. Pearson correlation significance codes above the diagonal can be interpreted as: ‘.’ 0.05 – 0.1; ‘*’ 0.01 – 0.5; ‘**’ 0.001 – 0.01; ‘***’ 0 – 0.001.


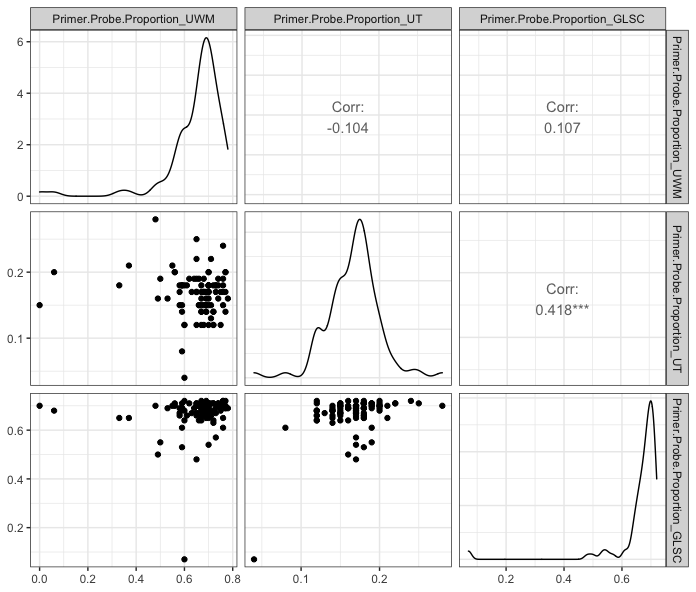


Fig. S8: Pairs-plot comparing *primer:probe* proportion identified for all 95 individuals sequenced using the GT-seq panel. Pearson correlation significance codes above the diagonal can be interpreted as: ‘.’ 0.05 – 0.1; ‘*’ 0.01 – 0.5; ‘**’ 0.001 – 0.01; ‘***’ 0 – 0.001.
